# Understanding Electrolyte Ion Size Effects on the Performance of Conducting Metal–Organic Framework Supercapacitors

**DOI:** 10.1021/jacs.4c00508

**Published:** 2024-04-26

**Authors:** Jamie W. Gittins, Kangkang Ge, Chloe J. Balhatchet, Pierre-Louis Taberna, Patrice Simon, Alexander C. Forse

**Affiliations:** † Yusuf Hamied Department of Chemistry, University of Cambridge, Lensfield Road, Cambridge CB2 1EW, U.K.; ‡ CIRIMAT, UMR CNRS 5085, Université Paul Sabatier Toulouse III, Toulouse 31062, France; § RS2E, Réseau Français sur le Stockage Electrochimique de l’Energie, FR CNRS 3459, Amiens Cedex 80039, France

## Abstract

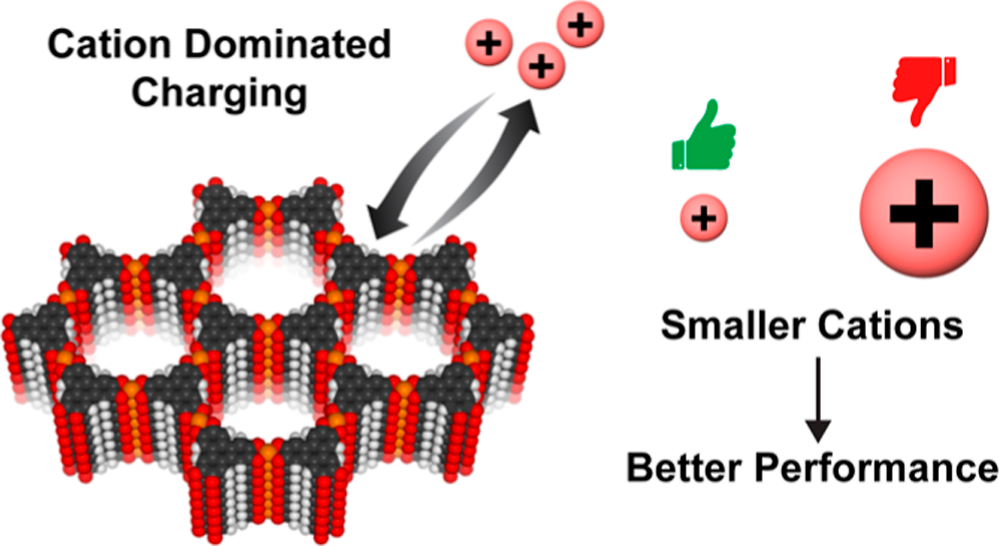

Layered metal–organic frameworks (MOFs) have emerged as promising materials for next-generation supercapacitors. Understanding how and why electrolyte ion size impacts electrochemical performance is crucial for developing improved MOF-based devices. To address this, we investigate the energy storage performance of Cu_3_(HHTP)_2_ (HHTP = 2,3,6,7,10,11-hexahydroxytriphenylene) with a series of 1 M tetraalkylammonium tetrafluoroborate (TAABF_4_) electrolytes with different cation sizes. Three-electrode experiments show that Cu_3_(HHTP)_2_ exhibits an asymmetric charging response with all ion sizes, with higher energy storage upon positive charging and a greater charging asymmetry with larger TAA^+^ cations. The results further show that smaller TAA^+^ cations demonstrate superior capacitive performances upon both positive and negative charging compared to larger TAA^+^ cations. To gain further insights, electrochemical quartz crystal microbalance measurements were performed to probe ion electrosorption during charging and discharging. These reveal that Cu_3_(HHTP)_2_ has a cation-dominated charging mechanism, but interestingly indicate that the solvent also participates in the charging process with larger cations. Overall, the results of this study suggest that larger TAA^+^ cations saturate the pores of the Cu_3_(HHTP)_2_-based electrodes. This leads to more asymmetric charging behavior and forces solvent molecules to play a role in the charge storage mechanism. These findings significantly enhance our understanding of ion electrosorption in layered MOFs, and they will guide the design of improved MOF-based supercapacitors.

## Introduction

In recent years, electrically conductive layered metal–organic frameworks (MOFs) have been synthesized with conjugated aromatic linker molecules.^1,2^ These materials, comprised of 2D π-d conjugated layers which stack to give a honeycomb structure, combine electrical conductivity with permanent porosity, making them promising electrode materials for energy storage applications.^3−8^ In particular, several layered MOFs have displayed encouraging capacitive performances in supercapacitors, underlining the potential of these frameworks in fast-charging energy storage devices.^9−14^ While recent work has attempted to improve the performances of MOF-based supercapacitors through optimization of particle morphology,^15−17^ more studies are needed to further maximize device performances and to better understand the electrochemical behavior of these emerging materials.^18^ Fortunately, the well-defined and tunable structures of MOFs open the door to structure–performance studies that are difficult to perform with traditional porous carbon electrodes, facilitating the optimization of MOF-based supercapacitors.

One design criterion that has not yet been optimized for layered MOF supercapacitors is the electrolyte ion size to electrode pore size ratio. Previous studies have attempted to answer this question for nanoporous carbon electrodes, although differences and difficulties in analyzing the pore structure of carbon materials, which have amorphous and disordered structures, have led to conflicting and confusing results.^19−26^ One effect observed in some studies on this topic is “porosity saturation” of the porous carbon electrode.^27^ This involves the complete filling of the electrolyte accessible surface area of the electrode during charging, and occurs while using electrolytes with larger ion sizes and carbons with low total porosities. This tends to result in asymmetric charging with a decreasing capacitance at high cell voltages due to saturation of the pores. These findings indicate that smaller ion sizes are favorable for higher performance when using electrode materials with lower total porosities than traditional state-of-the-art porous carbons. While the impact of electrolyte ion size on the performances of porous carbons has been studied in detail, layered MOF electrodes remain underexplored,^28^ with no studies looking at the impact of the ion size on the performances of triphenylene-based MOFs which have been widely studied in the literature and display primarily double-layer capacitive behavior. This study would provide insights into the ideal ion size for this family of materials, allowing for further optimization of MOF-based systems. The well-defined structures of MOFs would also help to test general design principles for supercapacitors more widely.

Design principles for improved supercapacitors should be underpinned by a fundamental understanding of the charging mechanisms. Such understanding provides deeper insights into why certain electrode and electrolyte combinations perform better than others and could inspire new routes to increased performance. To date, only a few computationally focused studies have examined the charging mechanisms of MOF-based supercapacitors, with the first such study modeling the double-layer structure and charging process of Ni-based layered MOFs with a variety of pore sizes using molecular dynamics (MD) simulations.^29,30^ More recently, Walsh et al. expanded on this by studying the electrochemical interface of the layered MOF Cu_3_(HHTP)_2_ (HHTP = 2,3,6,7,10,11-hexahydroxytriphenylene) with tetraethylammonium tetrafluoroborate (TEABF_4_) in acetonitrile electrolyte using a multiscale quantum-mechanics/molecular-mechanics (QM/MM) approach.^30^ In contrast to the previous MD approach, this method accounted for the heterogeneous electronic structure of the MOF electrode and revealed how it changes with charging. Excitingly, comparison of simulated capacitance values obtained for a range of different charging mechanisms with experimental results indicated that cations are the dominant species participating in charge storage for Cu_3_(HHTP)_2_, with minimal involvement of the anions. However, there has been a lack of experimental work to study the ion electrosorption in layered MOFs to confirm these findings.^31^ One technique that can be used to study ion adsorption during charging is electrochemical quartz crystal microbalance (EQCM), a technique that has been used extensively in nanoporous carbon and MXene devices to understand their charging mechanisms with a range of electrolytes.^32−36^ EQCM relies on measuring the change in resonance frequency of a Au-coated quartz crystal that is covered with a thin layer of active material to calculate the mass change of a working electrode in an electrochemical cell. By monitoring this mass change as the electrode is charged and discharged for a capacitive system, ion fluxes can be determined. This has been used to investigate the ion electrosorption process in supercapacitors, and the charge storage mechanisms in batteries.^37−39^ However, no studies have used EQCM to look at the double-layer charging of layered MOFs.^8,28,31,40^ Employing this technique to study the charge storage mechanisms of MOF-based devices alongside measurements of their electrochemical performance would allow the link between charging mechanisms and performance to be better understood.

Here, we present a detailed study examining the impact of electrolyte cation size on the capacitive performance and charge storage mechanism of the layered MOF Cu_3_(HHTP)_2_, which has a well-defined unimodal pore size distribution (PSD). We find that smaller cations result in higher capacitive performances upon both positive and negative charging, with highly asymmetric charging seen with larger cations. EQCM measurements suggest a cation-dominated charging mechanism for this series of tetraalkylammonium (TAA^+^) electrolytes, with the potential involvement of the solvent for electrolytes with larger cation sizes. Overall, our findings indicate that larger electrolyte cations lead to porosity saturation in the MOF electrodes, resulting in more asymmetric charging and lower overall energy storage performance. This is the first work to examine the charging mechanisms of a layered MOF supercapacitor using operando EQCM, and it demonstrates that smaller electrolyte ions lead to higher capacitive performances in MOF-based supercapacitors.

## Results and Discussion

### Material Synthesis and Characterization

**Figure 1:**
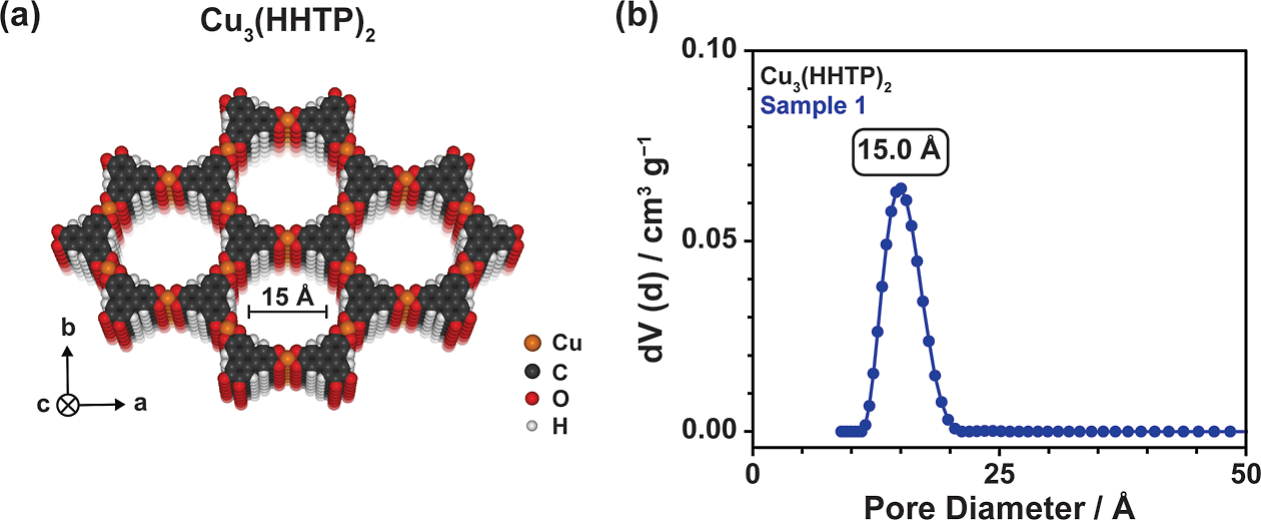
(a) Schematic showing the structure of the conductive layered MOF Cu_3_(HHTP)_2_, along with (b) the PSD of Cu_3_(HHTP)_2_ calculated from a N_2_ sorption isotherm using a N_2_ at 77 K on carbon (cylindrical pores) quenched solid density functional theory model. This shows the unimodal and well-defined pore structure of this framework, centered at approximately 15 Å. The 77 K N_2_ sorption isotherm for this sample is shown in Figure S3.

The layered MOF Cu_3_(HHTP)_2_ (Figure 1a) was chosen as the model electrode material for this study as it has a well-defined pore size and structure, and its electrochemical performance has been characterized in detail in previous work.^14,15^ Polycrystalline samples of Cu_3_(HHTP)_2_ were synthesized using a previously reported literature synthesis (see Supporting Information),^11^ and the chemical composition of the framework was confirmed by elemental analysis, which revealed the expected ratio of Cu and HHTP (Table S1). Elemental analysis also showed that some nitrogen-containing impurities remain in the framework after extensive washing and activation, consistent with previous reports.^14,41^ Scanning electron microscopy confirmed that the use of ammonia as a modulator in the synthesis resulted in the formation of flake-like crystallites, which were previously shown to be the crystal morphology that exhibits the best performance in Cu_3_(HHTP)_2_ supercapacitor devices (Figure S1).^15^ Two samples of Cu_3_(HHTP)_2_ (sample 1 and sample 2) were synthesized for this work and tested throughout to ensure that the results seen were consistent between different sample batches of the MOF. Characterization data for both samples are shown in the Supporting Information. Unless stated, all data shown in the main text are from sample 1.

Powder X-ray diffraction (PXRD) patterns confirmed the high crystallinity of as-synthesized Cu_3_(HHTP)_2_ (Figure S2) and indicated that this material has a hexagonal eclipsed unit cell with AA layer stacking. Unit cell parameters of *a* ≈ *b* = 21.2 Å and an interlayer spacing *c* = 3.1 Å were calculated from the PXRD patterns. This leads to a predicted crystallographic pore size of 16 Å. The PSD of this material, along with its porosity, was further analyzed using 77 K N_2_ sorption isotherms (Figure S3). Brunauer–Emmett–Teller (BET) analysis on two independent samples confirmed that the material synthesized in this work is highly porous, with a BET surface area of 802 ± 40 m^2^ g^–1^ obtained.^42^ Importantly, PSD analysis confirmed that Cu_3_(HHTP)_2_ has an ordered, unimodal PSD centered at 15.0 Å (Figure 1b), in good agreement with the crystallographic pore size. This further confirms the well-defined long-range order of this material. This is in contrast to porous carbons, which have significantly more disordered structures with little long-range ordering and broad pore size distributions (Figure S4). Overall, this supports the use of layered MOFs, such as Cu_3_(HHTP)_2_, to establish structure–performance relationships in supercapacitors.

PXRD and N_2_ sorption analysis confirmed that the long-range order and porosity of Cu_3_(HHTP)_2_ is largely maintained upon forming a composite electrode film with PTFE and a conductive additive for use in electrochemistry experiments (Figures S5 and S6).^14^ However, the N_2_ sorption analysis reveals a decrease in the porosity of Cu_3_(HHTP)_2_ in the electrode films, with a BET area of 545 ± 7 m^2^ g^–1^ calculated for the constituent Cu_3_(HHTP)_2_ after removing the contribution of acetylene black, and assuming PTFE does not contribute to N_2_ adsorption. This represents a decrease in the surface area of Cu_3_(HHTP)_2_ of approximately 32% compared to that of powder samples. Furthermore, there is a slight reduction in the modal pore size to 14–14.2 Å. This shows that PTFE and/or acetylene black are blocking some of the pores and reducing the available surface area of the MOF. While infiltration of polymers into the pores of MOFs has been observed previously using nuclear magnetic resonance (NMR),^43^ this effect had not previously been quantified for conductive layered MOFs. Although a decrease in porosity has also been observed for porous carbon materials when made into composite electrode films, the percentage decrease in surface area was much smaller.^44,45^ This was confirmed in this work, where a decrease of 4% in the BET area of YP80F was observed upon formation of an electrode film using the same method as above for Cu_3_(HHTP)_2_ (Figure S7). This suggests that the binder and conductive additive are significantly more effective at blocking the ordered one-dimensional porosity in layered MOFs. Optimization of layered MOF composite electrode films, by changing both the conductive additive and binder, may help us to minimize this effect and improve the performances of these materials in supercapacitors. Having confirmed the well-defined structure of Cu_3_(HHTP)_2_, we proceeded to use this layered MOF to probe the cation size–performance relationship.

### Three-Electrode Energy Storage Measurements of Cu_3_(HHTP)_2_

**Figure 2:**
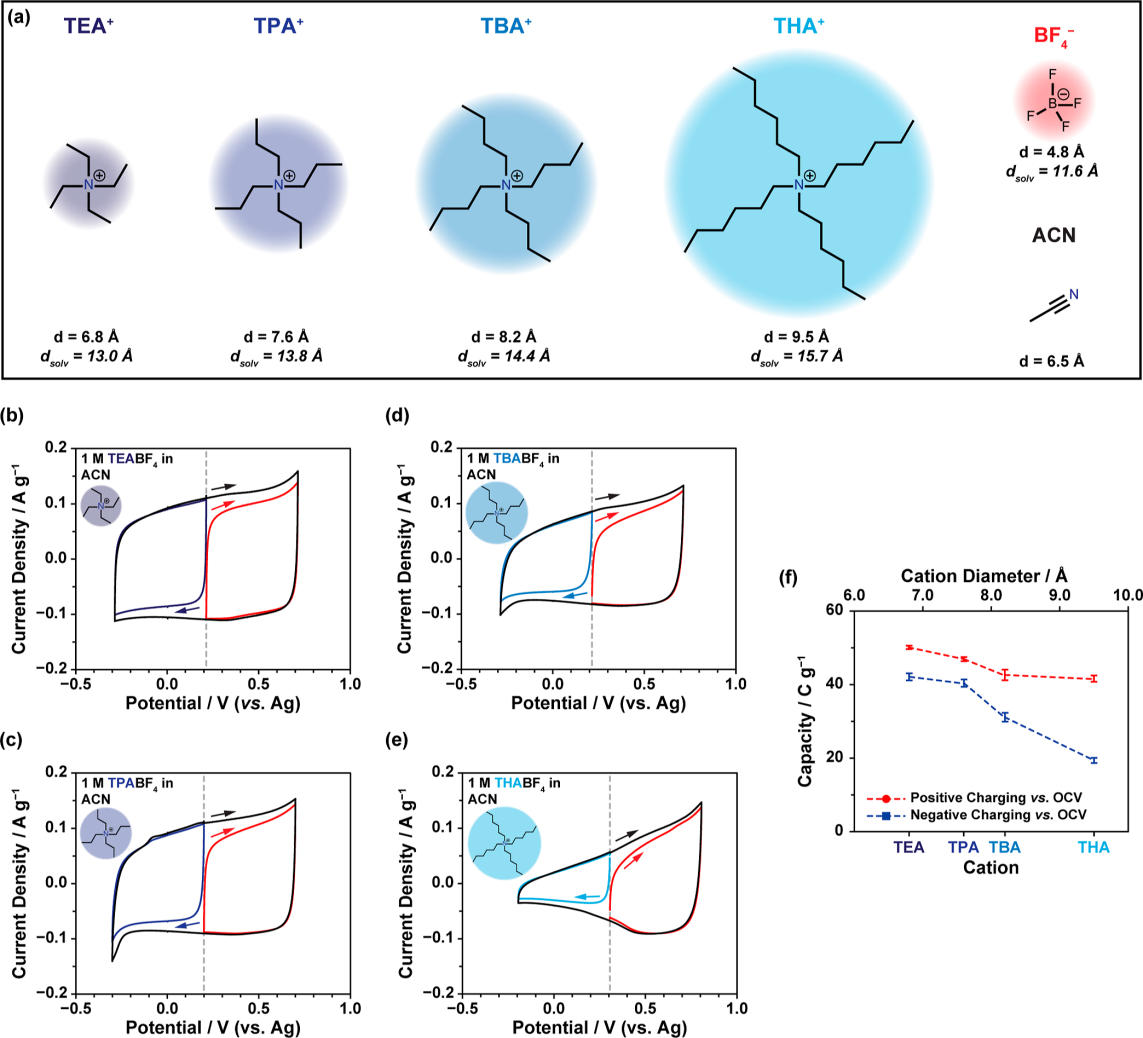
(a) Lewis structure of each tetraalkylammonium (TAA^+^) cation, along with the naked and solvated ion sizes, for each electrolyte species used in this study. The solvated ion sizes are calculated assuming a primary solvation shell of acetonitrile molecules.^46−49^ (b–e) Cyclic voltammetry (CV) data obtained at a scan rate of 1 mV s^–1^ from three-electrode cells assembled with Cu_3_(HHTP)_2_ working electrodes, YP80F oversized counter electrodes, and Ag pseudoreference electrodes, with 1 M solutions of (b) TEABF_4_, (c) TPABF_4_, (d) TBABF_4_, and (e) THABF_4_ in acetonitrile as electrolytes. The open circuit potential (OCP) is indicated by a dashed line. Data were acquired by scanning to +0.5 V vs OCP (positive charging; red), −0.5 V vs OCP (negative charging; blue), and across the full potential window (black). Cu_3_(HHTP)_2_ was stable and showed minimal Faradaic peaks between the chosen potential limits for each electrolyte, confirming mainly double-layer type charge storage for each electrolyte in this potential window. The direction of scanning is indicated by the arrow in each case. (f) Specific capacity values calculated from galvanostatic charge–discharge (GCD) profiles from three-electrode cells at a current density of 0.05 A g^–1^ when charging to +0.5 V vs OCP (red) and −0.5 V vs OCP (blue). The red and blue dashed lines are added as guidelines. The mass of Cu_3_(HHTP)_2_ in the working electrode was used to calculate the specific capacity values. This shows how the charge storage changes with the cation size for both positive and negative charging.

The impact of ion size on the energy storage performance of Cu_3_(HHTP)_2_ was studied by using a series of 1 M tetraalkylammonium tetrafluoroborate (TAABF_4_) in acetonitrile electrolytes (Figure 2). While all electrolytes had the same anion (BF_4_^–^), and thus a constant anion size (4.8 Å naked ion size; 11.6 Å solvated ion size),^50^ the TAA^+^ cation was varied to systematically change the cation size. In total, four different cations were used: tetraethylammonium (TEA^+^), tetrapropylammonium (TPA^+^), tetrabutylammonium (TBA^+^), and tetrahexylammonium (THA^+^), with their naked and solvated ion sizes summarized in Figure 2a. This allowed for a variation in unsolvated cation size between 6.8–9.5 Å (solvated: 13.0–15.7 Å).^50,51^ A three-electrode arrangement enabled the response of Cu_3_(HHTP)_2_ upon both negative and positive charging relative to the open cicuit potential (OCP) to be studied separately (Figures 2b–e and S8). To evaluate the charge storage, both specific capacity and specific capacitance were calculated from galvanostatic charge–discharge (GCD) experiments for both positive and negative charging from three-electrode cells (Figure 2f; Tables S2 and S3; and Figure S9). All experimental capacity and capacitance values for Cu_3_(HHTP)_2_ were calculated after removing the capacitive contribution of acetylene black that is also present in the electrodes, and all values were normalized to the mass of Cu_3_(HHTP)_2_ in the working electrode. Capacity is reported as the primary energy storage performance metric in this work rather than capacitance due to the nonlinearity of the GCD discharge curves produced from these systems. This is in accordance with previous recommendations on data analysis.^52^ Low current densities and scan rates were used to attempt to ensure complete permeation of the ions into the MOF pores and to minimize the impact of differences in ion kinetics on the results.

Both CV and GCD three-electrode experiments reveal exciting insights into the cation size–performance behavior of Cu_3_(HHTP)_2_. First, an overall decrease in capacity for both positive and negative charging was observed as the solvated electrolyte cation size approaches and then exceeds the electrode pore size of approximately 14 Å (Figure 2f), with TEA^+^ having the highest capacity for both positive and negative charging. This result demonstrates that smaller electrolyte ions lead to higher energy storage performances in MOF-based supercapacitors and represents the first time that the impact of ion size on the performances of triphenylene-based conductive layered MOFs has been uncovered. One potential explanation for this cation size–performance behavior is that the nitrogen charge center of the smaller TAA^+^ cations can get closer to the pore walls of Cu_3_(HHTP)_2_, resulting in smaller charge separation distances and higher capacities.^19,20^ These results confirm, as expected, that electrolytes with smaller ion sizes should be employed in conductive layered MOF supercapacitors in the future to optimize their energy storage performances.

The CV data also show that Cu_3_(HHTP)_2_ displays a clear asymmetric charging response with all TAA^+^ electrolytes, with the capacity upon positive charging greater than the capacity upon negative charging (Figure 2b–e). It is worth noting that there is an increase in the degree of asymmetry as the cation size increases, with a significant increase from TPA^+^ to TBA^+^, and THA^+^ having the greatest difference in capacity between positive (41.6 ± 1.4 C g^–1^) and negative (19.5 ± 1.4 C g^–1^) charging. Interestingly, this charging behavior is reminiscent of porosity saturation that has been observed previously in low porosity carbon electrodes with electrolytes with large cations.^27^ These systems also exhibit asymmetric charging with a significant decrease in charge storage at high cell voltages as the carbon pores become saturated by large TAA^+^ cations during charging. Porosity saturation in Cu_3_(HHTP)_2_ is plausible due to the relatively low total porosities of the MOF electrode films compared to other traditional supercapacitor electrode materials, such as porous carbons.^30,44,45^ While there is only a small decrease in the negative charging capacity when the cation size is increased from TEA^+^ (42.1 ± 1.9 C g^–1^) to TPA^+^ (40.4 ± 2.0 C g^–1^), there is a much larger drop in capacity between TPA^+^ and TBA^+^ (31.2 ± 2.0 C g^–1^). This strongly suggests that porosity saturation becomes more significant for cation sizes of TBA^+^ and above, which provides another explanation for the higher charge storage performances of smaller cations. Three-electrode rate capability and electrochemical impedance spectroscopy (EIS) measurements support this hypothesis. Both measurements indicate poorer kinetic performance and decreases in ion mobility as the cation size increases, consistent with increasing pore saturation with larger TAA^+^ cations (Figures S9–S11; Table S3). The observed differences in charging kinetics in this work are consistent with recent experimental investigations, which indicate slower charging kinetics in systems with smaller pore sizes and a fixed electrolyte size, equivalent to fixing the pore size and increasing the ion size as done here.^53−55^ Ion sieving, a related phenomenon which has been seen in other layered materials, also gives rise to similar asymmetric charging behavior,^49,56,57^ but is unlikely in these systems as the naked (desolvated) cation sizes are all significantly smaller than the electrode pore size.

Electrochemical experiments on symmetric two-electrode supercapacitors assembled with this series of electrolytes support the above findings (Figures S12–S14; Table S4). Once again, TEA^+^ showed the best energy storage performance with Cu_3_(HHTP)_2_, supporting the conclusion that smaller electrolyte ions are favorable for high capacitive performances with this class of materials. In addition, a large drop in performance was seen between TPA^+^ and TBA^+^, providing further evidence for porosity saturation with larger TAA^+^ cations (TBA^+^ and THA^+^). The asymmetric charging behavior of these systems has additional consequences for symmetric two-electrode cells. For electrolytes with larger cations, differences in capacity between the two oppositely polarized electrodes results in the potential of the negative electrode going outside its stable potential window. This leads to quasi-reversible Faradaic activity at lower cell voltages, resulting in more facile degradation of Cu_3_(HHTP)_2_ and reducing the energy density of devices with larger cations (Figure S12). This further highlights the superior performance of smaller TAA^+^ cations (TEA^+^ and TPA^+^) with Cu_3_(HHTP)_2_. To assess whether changing the anion size impacts the electrochemical performance of Cu_3_(HHTP)_2_, two-electrode measurements were performed with both 1 M tetraethylammonium tetrafluoroborate (TEABF_4_) and 1 M tetraethylammonium bis(trifluoromethanesulfonyl)imide (TEATFSI) in acetonitrile electrolytes (Figure S15). These electrolytes have the same cations (TEA^+^) but have anions of different sizes, with TFSI^–^ (7.9 Å naked ion size along the longest dimension) having a larger ion size compared to BF_4_^–^ (4.8 Å naked ion size).^21^ These experiments indicate that there is no clear effect of the anion on the charge storage performance with similar specific capacitances recorded with both electrolytes at a low current density (116 F g^–1^ for BF_4_^–^ compared to 121 F g^–1^ for TFSI^–^). However, further studies with a wider range of anions are required to confirm this.

To investigate the results with different TAA^+^ cations in more detail and better understand the impact of cation ion size on the performance of Cu_3_(HHTP)_2_, EQCM was used to probe the ion flux and charging mechanisms of these systems.

### EQCM Studies of the Charging Mechanism

**Figure 3:**
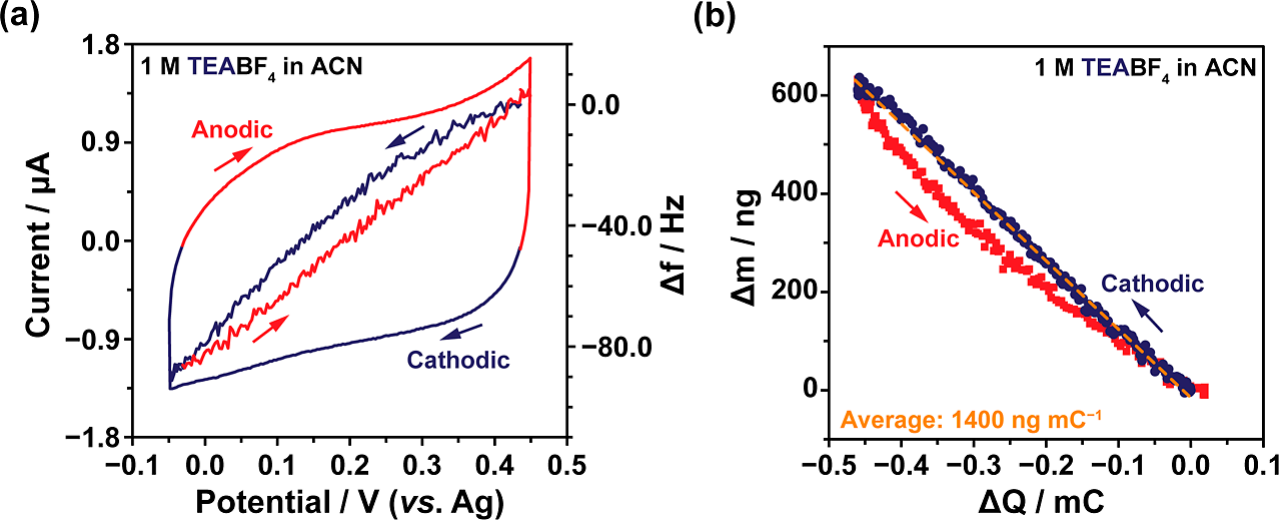
(a) CV and EQCM frequency response of Cu_3_(HHTP)_2_ with 1 M TEABF_4_ in acetonitrile electrolyte, obtained at a scan rate of 1 mV s^–1^ in the potential range from −0.05 to +0.45 V vs Ag. This potential range was chosen to avoid non-capacitive processes that occur on the quartz surface at low scan rates. All EQCM cells were assembled with Cu_3_(HHTP)_2_-coated quartz working electrodes, platinum wire counter electrodes, and Ag pseudoreference electrodes. (b) Plot of electrode mass change, calculated from the frequency response shown in (a), against accumulated charge (Δ*Q*). Δ*Q* was calculated by integrating the current against time for the CV, and setting the charge at the electrode potential of +0.45 V vs Ag to zero. The frequency response and mass change are considered separately for cathodic (blue) and anodic (red) polarizations. The dashed line (orange) shows the average mass change during the full CV experiment. The OCP of the cell used to produce the data in the figure above is +0.16 V vs Ag.

EQCM studies were initially performed on Cu_3_(HHTP)_2_ with 1 M TEABF_4_ in an acetonitrile electrolyte (Figure 3). The CV was subdivided into two sections based on the polarization of the electrode: a cathodic regime with a negative current response (*I* < 0) and an anodic regime with a positive current response (*I* > 0) (Figure 3a; outside lines). EQCM cells with TEA^+^ were cycled between potential limits of +0.29 V vs OCP to −0.21 V vs OCP. Although the potential window is narrower than that used in the three-electrode experiments above, it covers both positive and negative charging as defined for Figure 2b, allowing the charging mechanism to be studied for both charging directions. The lower stable potential window of the EQCM cell was to avoid any potential instabilities of the cell materials and electrolyte at the low scan rate (1 mV s^–1^) used in this work.^58^ Also shown in Figure 3a (inside lines) is the change in the resonance frequency response, Δ*f*, of the Cu_3_(HHTP)_2_-coated quartz crystal during the CV experiment. In this work, Sauerbrey’s equation applies as there was negligible change in motional resistance (Δ*R*) during CV cycling for the EQCM cells (Figures S16 and S18). As a result, Δ*f* of the Cu_3_(HHTP)_2_-coated quartz crystal was translated into a mass change (Δ*m*) of the working electrode during the cycling, where an increase in Δ*m* corresponds to a decrease in Δ*f* (see Supporting Information for details).^59^ Δ*m* was then plotted against the accumulated electronic charge, Δ*Q*, (Figure 3b) to allow analysis of the charging mechanism in greater detail, including assigning the different species entering and leaving the electrode pores during charging and discharging. If Δ*m* is negatively correlated with Δ*Q* for cathodic charging, cations (“counterions”) are the main charge carriers, while the opposite stands for a positive correlation.^60^

**Figure 4:**
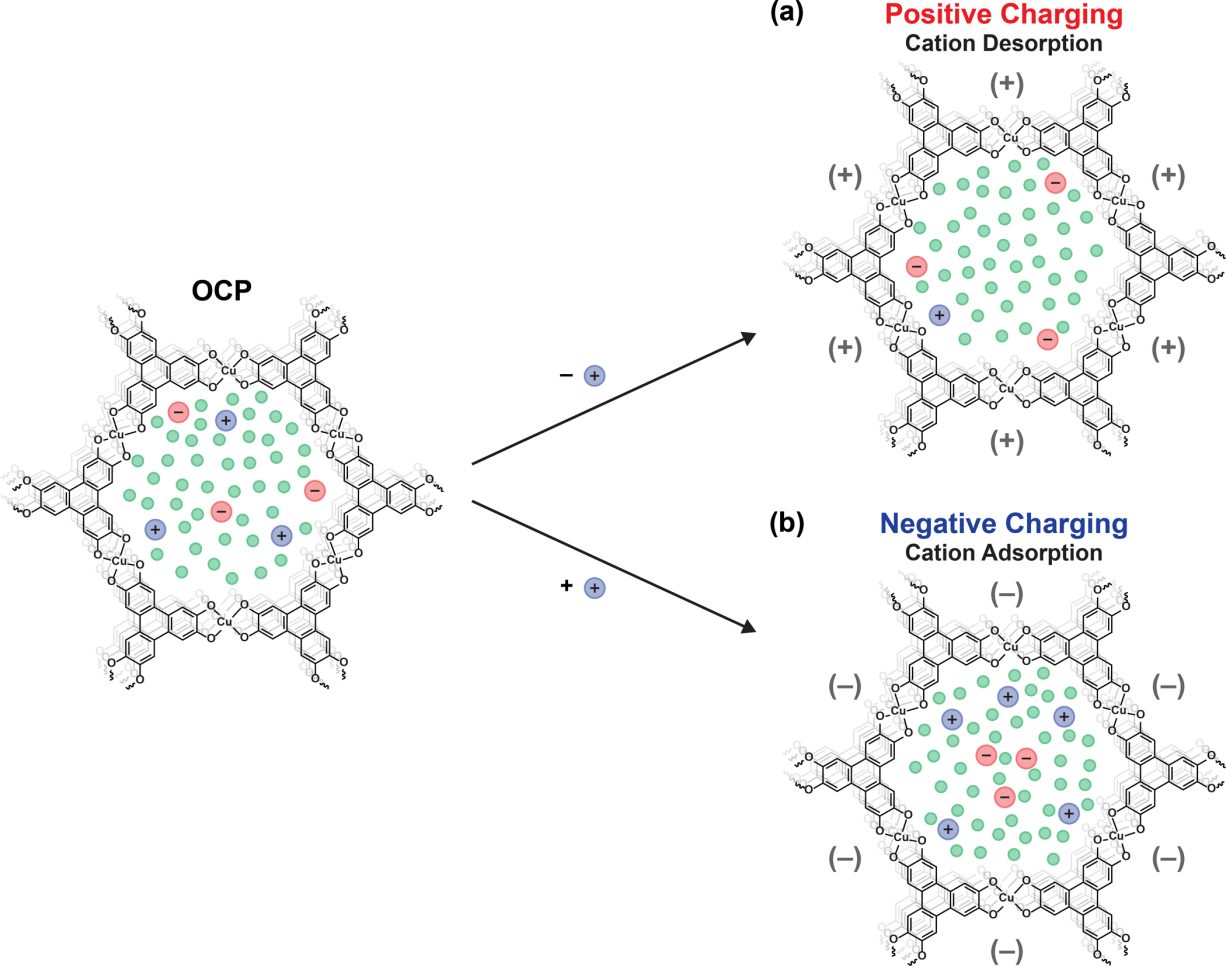
Cartoon of the cation-dominated charging mechanism of CuHHTP with 1 M TEABF_4_ in acetonitrile, as deduced from EQCM and computational studies.^30^ (a) Upon positive charging, there is cation desorption from the MOF pores, with the anions shifting to a more strongly interacting edge adsorption site. (b) Upon negative charging, the reverse occurs with cation adsorption and corresponding movement of the anions to a more weakly bound center adsorption site. Note that the ion and solvent sizes are not to scale, and the solvation of electrolyte ions is not shown.

TEABF_4_ showed an approximately rectangular EQCM CV response, consistent with the three-electrode experiments described above (Figure 3a). The Δ*m*–Δ*Q* plot for this system demonstrates a reversible and linear mass change during cycling, with the mass change upon both cathodic (negative) and anodic (positive) charging aligning closely with the average value (dashed orange line, Figure 3b). This suggests a kinetically reversible process with the same dominant charge carrier for both charging directions. As the slope of the Δ*m*–Δ*Q* plot is negative, this indicates that cation adsorption and desorption are the dominant charge storage mechanisms. If anions were involved in the charging mechanism, as would be the case for an ion exchange mechanism, there would be an obvious decrease in the slope of the Δ*m*–Δ*Q* plot with charge accumulation, resulting in a weaker Δ*m*–Δ*Q* correlation. Both the cathodic and anodic charging regimes show an average mass change close to 1400 ng mC^–1^, corresponding to an equivalent molecular weight of 139 ± 4 g mol^–1^ (Figure S17). This aligns closely with the molecular weight of the naked TEA^+^ ion (130 g mol^–1^) and further confirms that the cations are the dominant charge carriers across the full potential range considered here. As the experimental mass change is consistent with the adsorption and desorption of naked TEA^+^ cations, this charging mechanism would require no net movement of anions or solvent molecules in or out of the electrode pores. However, the solvated ion size of TEA^+^ (13.0 Å) in the bulk electrolyte^27^ is less than the average pore size of Cu_3_(HHTP)_2_ in the electrode film (∼14 Å), indicating that the solvated TEA^+^ cations are still able to enter and leave the pores of the MOF. If this were to occur during the charging mechanism, an equivalent amount of free solvent molecules would need to leave or enter the pores. This demonstrates a key limitation of EQCM experiments, as the contributions of different electrolyte species to the net mass change cannot be distinguished, making it difficult to determine the solvation state of the TEA^+^ cations during cation adsorption and desorption. Overall, however, our EQCM results suggest that cation desorption (positive charging) and cation adsorption (negative charging) are the primary charging mechanisms for Cu_3_(HHTP)_2_ in 1 M TEABF_4_ in the potential range from −0.05 to +0.45 V vs Ag (Figure 4). This is in contrast to the double-layer charge storage mechanism of traditional porous carbons, where both the anions and cations have been shown to play a role in the charging mechanisms.^32,34,61^ This finding also begins to explain our capacity data in Figure 2b–e, where variation in the size of the cation (i.e., the dominant charge carrier) impacted the capacitance for *both* positive and negative charges in the present potential window. Excitingly, this finding is in line with recent computational work on this system, which suggested a cation-dominated charging mechanism for this system, with good agreement between the simulated areal capacitance values calculated for a cation-dominated charging process and those obtained from the three-electrode experiments with TEA^+^ in this work (Table S5).^30^

Although the EQCM results suggest that the anions are not involved in the charging mechanism, their involvement cannot be ruled out. Simulations from Walsh et al. found that TEA^+^ cations only have one possible adsorption site in the pores of Cu_3_(HHTP)_2_, while BF_4_^–^ anions have two adsorption environments: a weaker binding site at the center of the pore and a stronger binding site at the pore edge where the BF_4_^–^ anions interact with the C–H moieties of the HHTP linker molecules (Figure 4).^30^ At the OCP, the simulations showed that anions exist in both environments. However, upon positive charging (cation desorption), simulations found that the anions shift and predominantly occupy the more strongly bound edge site. Upon negative charging (cation adsorption), the opposite occurs with the anions shifting to occupy the pore center site. However, as there is no net change in the in-pore population of BF_4_^–^ from this process, and thus no net anion flux, EQCM is not able to detect this potential contribution of the anions to the charging mechanism, although it is likely to be a more minor contribution to the overall charge storage compared to the adsorption/desorption of cations. This illustrates that a multitechnique approach is required to get a complete picture of the charge storage mechanism. Other experimental techniques, such as NMR spectroscopy, should be used to better understand the role of both the anions and solvent in the charging mechanism of this system.^62,63^

**Figure 5:**
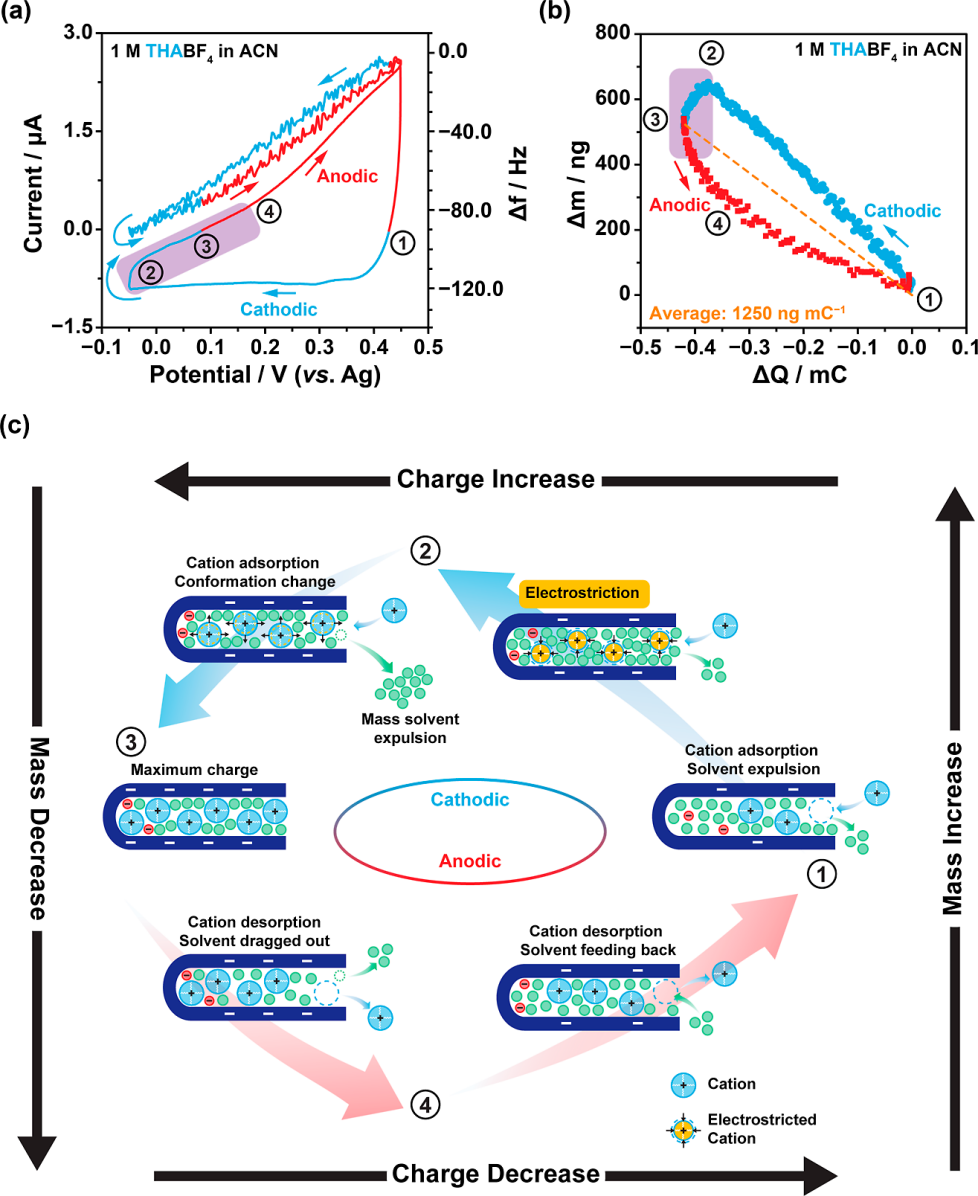
(a) CV and EQCM frequency response of Cu_3_(HHTP)_2_ with 1 M THABF_4_ in acetonitrile electrolyte, obtained at a scan rate of 1 mV s^–1^ in the potential range from −0.05 to +0.45 V vs Ag. All EQCM cells were assembled with Cu_3_(HHTP)_2_-coated quartz working electrodes, platinum wire counter electrodes, and Ag pseudoreference electrodes. (b) Plot of electrode mass change, calculated from the frequency response shown in (a), against accumulated charge. The frequency response and mass change are considered separately for positive (cathodic, shown in blue) and negative (anodic, shown in red) polarizations. The dashed line (orange) shows the average mass change during the full CV experiment, calculated from Faraday’s law. The abnormal mass drop is highlighted in violet. The OCP of the cell used to produce the data in the figure is +0.22 V vs Ag. (c) Cartoon schematic of the possible charging mechanism of Cu_3_(HHTP)_2_ with 1 M THABF_4_, showing cation and solvent movement upon cathodic charging and anodic discharging. Note that the solvation of the electrolyte ions is not shown.

Building on the results with TEABF_4_, EQCM studies were also performed with 1 M THABF_4_ in acetonitrile electrolyte to assess how the charging mechanism changes upon increasing the cation size, and to provide insights into why larger cations lead to lower energy storage performances with Cu_3_(HHTP)_2_ (Figure 5). Negligible changes in motional resistance (Δ*R*) were also observed with this electrolyte (Figure S18). EQCM cells with THA^+^ were cycled between the potential limits of +0.23 V vs OCP to −0.27 V vs OCP, allowing for investigation of the charge storage mechanism for both positive and negative charging, as defined for Figure 2f. As observed in the 3-electrode measurements, THA^+^ exhibited an asymmetric CV (Figure 5a), resulting in an asymmetric Δ*m*–Δ*Q* plot that displays a clear mass hysteresis about the average mass change of 1250 ng mC^–1^ (dashed orange line; Figure 5b). This indicates clear differences in the charging mechanism between the cathodic and anodic charging regimes (Figure 5b), in contrast to the results for TEA^+^ (Figure 3b).

During the cathodic scan from Δ*Q* = 0 to Δ*Q* = −0.38 mC, there is an approximately linear increase in Δ*m* with an average slope of 1660 ng of mC^–1^ (Figure 5b). However, the equivalent molecular weight corresponding to this mass increase was calculated to be only 163 ± 3 g mol^–1^ (Figure S19), less than the molecular weight of the naked THA^+^ cation (298 g mol^–1^). If this was due to involvement of anions in the charging process, a parabolic-like Δ*m*–Δ*Q* plot would be expected, with an increasing slope as the total charge increases.^64^ However, as the mass change is approximately linear, and there is a negative correlation with Δ*Q*, it is likely that the anions do not play a significant role in the charge storage mechanism, and cations are once again the dominant charge carriers. Instead, involvement of the solvent in the charging process is a more likely explanation for the lower observed mass change, and this is consistent with a linear Δ*m*–Δ*Q* plot. Therefore, the results suggest that adsorption of THA^+^ cations is accompanied by a net loss of solvent molecules from the electrode pores during cathodic charging, with adsorption of one THA^+^ cation accompanied by the net loss of 3.3 acetonitrile molecules on average (Figure 5c; process from stage 1 to stage 2). Unfortunately, the exact solvation state of the THA^+^ cations upon adsorption cannot be deduced from EQCM. This observation supports the hypothesis of porosity saturation in Cu_3_(HHTP)_2_ when charging with larger TAA^+^ cations, with a net expulsion of solvent molecules from the pores required following cation adsorption upon cathodic charging, resulting in lower observed equivalent molecular weight changes. The impact of increasing the potential window on the cathodic charging mechanism was also explored by gradually increasing the positive potential limit from +0.2 V vs Ag to +0.6 V vs Ag (Figure S20). A consistent negative correlation between Δ*m* and Δ*Q* was observed between different potential windows, suggesting a constant and potential-window-independent cation-dominated charge storage mechanism.

Porosity saturation is further supported by examining the mass change in the potential range from −0.05 to +0.22 V vs Ag. In this region, a steep mass drop is observed (purple box; Figure 5b). This behavior is not seen with TEA^+^, and it is once more indicative of overcrowding of the electrode pores with big THA^+^ cations. One potential explanation for this unexpected mass drop is a conformational change in the large THA^+^ cations upon a change in polarization at high accumulated charges. At the start of the cathodic scan (Figure 5c; stage 1), THA^+^ cations with long flexible alkyl chains start to accumulate in the pores, resulting in a significant increase in the in-pore cation density. As a result, the tightly packed THA^+^ cations become electrostricted at high negative polarizations. However, when the polarization is reversed at −0.05 V (Figure 5c; stages 2 to 3), the release of electrostatic force drives a change in conformation of the electrostricted THA^+^ cations from a compact packing to a looser packing arrangement. A similar behavior is observed in surface-active ionic liquids with analogous structures.^65−67^ This conformational change is accompanied by an increase in the volume of the in-pore cations, resulting in an expulsion of solvent molecules from the saturated MOF pores and a sudden mass decrease. At the maximum accumulated charge (Figure 5c; stage 3), the in-pore cation population reaches a maximum and the in-pore solvent number reaches a corresponding minimum. This results in a highly concentrated in-pore ion population, leading to considerable steric hindrance between the long alkyl chains of THA^+^ and thus slower ion kinetics, evidenced by the resistive current response from stage 2 to 4 (purple box; Figure 5a). Poorer kinetic performance with THA^+^ is also seen in both rate capability and EIS measurements on three-electrode cells (Figures S9 and S10), supporting lower ion mobility of the larger TAA^+^ cations.

At the start of anodic charging (Figure 5c, stages 3 to 4), desorption of THA^+^ cations occurs and is accompanied by loss of solvent molecules associated with the cations. This leads to a pronounced observed mass loss in this region (Figure 5b). However, as anodic charging continues and more desorption of large THA^+^ cations from the MOF pores occurs, the amount of free in-pore volume significantly increases. As a result, solvent molecules can feed back into the electrode pores from the bulk electrolyte as cation desorption continues (Figure 5c; stages 4 to 1). This leads to a gradual reduction in the slope of the Δ*m*–Δ*Q* plot in this region (Figure 5b). Due to this phenomenon, there is a corresponding improvement in ion kinetics between stages 4 and 1, evidenced by the increased anodic current in this region (Figure 5a). However, as for TEA^+^, the possibility of anion involvement in the charging mechanism with THA^+^ cannot be fully ruled out, as EQCM gives only a net mass change and cannot distinguish between different species.

To investigate the charge storage mechanism of Cu_3_(HHTP)_2_ with an intermediate cation size between TEA^+^ and THA^+^, EQCM studies were also performed with 1 M TPABF_4_ in acetonitrile electrolyte (Figures S21 and S22). The Δ*m*–Δ*Q* plot with TPA^+^ once again showed a reversible and linear mass change with accumulated charge, with the negative correlation between Δ*m* and Δ*Q* supporting cation adsorption (cathodic charging) and cation desorption (anodic charging) charge storage mechanisms with this electrolyte. Interestingly, an average molecular weight change of approximately 165 g mol^–1^ is calculated from the slope of the Δ*m*–Δ*Q* plot. This is slightly lower than the molecular weight of the naked TPA^+^ cation (187 g mol^–1^) and may indicate net movement of solvent during the charging process, with a small loss of solvent from the pores of the Cu_3_(HHTP)_2_ working electrode during negative charging (cation adsorption) and vice versa for positive charging (cation desorption). Excitingly, this behavior lies between that observed for TEA^+^, where no evidence for net solvent movement was observed, and THA^+^, where there was significant participation of solvent in the charging mechanism (Table 1). This supports the hypothesis that there is increasing participation of the solvent in the charge storage mechanism as the cation size increases, likely due to an increasing degree of porosity saturation in Cu_3_(HHTP)_2_ when charging with larger TAA^+^ cations.

**Table 1:** Difference between the Theoretical Molecular Weight Change Assuming Pure Cation Adsorption upon Cathodic Charging and the Average Molecular Weight Change Observed for Cathodic Charging during EQCM Measurements^a^

Cation	Theoretical molecular weight change (pure cation adsorption)/g mol^–1^	EQCM observed molecular weight change/g mol^–1^
TEA^+^	130	139
TPA^+^	187	165
THA^+^	298	163

a The increasing difference between the two values as the cation size increases is indicative of greater involvement of solvent in the charging mechanism.

In summary, the EQCM results illustrate that the charging mechanism significantly changes as the cation size is increased from TEA^+^ to THA^+^. Furthermore, these results support the hypothesis from electrochemical measurements of porosity saturation in Cu_3_(HHTP)_2_ when charging with larger TAA^+^ cations, with a significantly lower available pore volume following cation adsorption, forcing the solvent to participate in the charge storage mechanism. This surprising observation has not been observed in previous supercapacitor systems. Together with the three-electrode measurements, this demonstrates that porosity saturation can occur in Cu_3_(HHTP)_2_ electrodes with large electrolyte ions, resulting in both asymmetric charging and lower capacities. This is likely related to the comparatively lower total pore volume of layered MOF composite films compared to those of most traditional porous carbons. As a result, electrolytes with small ion sizes should be used to maximize the performance of MOF-based supercapacitors. Additionally, this work also demonstrates the crucial significance of operando EQCM in elucidating ion transport mechanisms under nanoconfinement.

## Conclusions

To conclude, this study has probed both the ion-size performance relationship and the charging mechanism of the layered MOF Cu_3_(HHTP)_2_ to provide a detailed description of the electrochemical behavior of this material. Three-electrode measurements revealed that smaller cations result in higher charge storage performances upon both positive and negative charging, showing that electrolytes with small ion sizes should be employed in layered MOF supercapacitors to maximize their performances. Furthermore, our results indicate that porosity saturation of the Cu_3_(HHTP)_2_ electrode occurs while using electrolytes with larger cations. Charging mechanism studies with EQCM support these findings. These measurements revealed that Cu_3_(HHTP)_2_ exhibits a cation-dominated charging mechanism with TEABF_4_, the electrolyte with the smallest cation used in this study, with co-ion desorption upon positive charging and counterion adsorption upon negative charging. No net movement of the solvent or anions in or out of the electrode pores was observed for this system. However, with THABF_4_, EQCM indicated that solvent molecules also likely participate in the charging mechanism, illustrating that increasing the cation size changes the charging mechanism of MOF-based supercapacitors. This result further supports saturation of the MOF pores with larger electrolyte ions and confirms that electrolytes with smaller ion sizes should be targeted to optimize the performances of MOF-based devices going forward. Our findings on this crystalline electrode system with unimodal porosity also validate the previous results which demonstrated that porosity saturation can occur in electrode materials with low overall total porosities relative to state-of-the-art porous carbons. We envisage that our study will guide the design of improved supercapacitors in the future.

## Data Availability

All raw experimental data files are available in the Cambridge Research Repository, Apollo, with the identifier DOI: 10.17863/CAM.105384.

## Supporting Information Available

Additional experimental details, materials, methods, and additional discussion of EQCM analysis (PDF)
