# Supplementary material for: Understanding Electrolyte Ion Size Effects on the Performance of Conducting Metal–Organic Framework Supercapacitors
Source: J Am Chem Soc. 2024 Apr 26;146(18):12473–84. doi: 10.1021/jacs.4c00508 (PMC11082900; doi:10.1021/jacs.4c00508)
Supplement: Supplementary file 1 — ja4c00508_si_001.pdf [file ja4c00508_si_001.pdf]

# Supplementary Information for:

## Understanding Electrolyte Ion Size Effects on the Performance of Conducting Metal-Organic Framework Supercapacitors

Jamie W. Gittins[1,‡], Kangkang Ge[2,‡], Chloe J. Balhatchet[1], Pierre-Louis Taberna[2,3], Patrice Simon[2,3*], Alexander C. Forse[1*]

[1] Yusuf Hamied Department of Chemistry, University of Cambridge, Lensfield Road, Cambridge CB2 1EW, UK.

[2] CIRIMAT, UMR CNRS 5085, Université Paul Sabatier Toulouse III, Toulouse 31062, France.

[3] RS2E, Réseau Français sur le Stockage Electrochimique de l'Energie, FR CNRS, 3459, Amiens Cedex 80039, France.

[‡] These authors contributed equally.

[*]Corresponding authors' emails: patrice.simon@univ-tlse3.fr, acf50@cam.ac.uk

# Contents


Methods & Materials ........ 3
Materials ........ 3
Synthesis ........ 3
Materials Characterisation ........ 3
Electrode Film Preparation ........ 4
Electrochemical Measurements ........ 4
SI Table S1 ........ 6
SI Figure S1 ........ 7
SI Figure S2 ........ 8
SI Figure S3 ........ 9
SI Figure S4 ........ 10
SI Figure S5 ........ 11
SI Figure S6 ........ 12
SI Figure S7 ........ 13
SI Figure S8 ........ 14
SI Table S2 ........ 15
SI Figure S9 ........ 16
SI Table S3 ........ 17
SI Figure S10 ........ 18
SI Figure S11 ........ 19
SI Figure S12 ........ 20
SI Figure S13 ........ 21
SI Table S4 ........ 22
SI Figure S14 ........ 23
SI Table S5 ........ 24
SI Figure S15 ........ 25
SI Figure S16 ........ 26
SI Figure S17 ........ 27
SI Figure S18 ........ 28
SI Figure S19 ........ 29
SI Figure S20 ........ 30
SI Figure S21 ........ 31
SI Figure S22 ........ 32
SI Additional Details ........ 33
Details of EQCM Calculations ........ 33

References ........................................................................................................................ 34

# Methods & Materials

## Materials

All materials were purchased from commercial suppliers, used without additional modification and handled in air unless specified below.

Tetraethylammonium tetrafluoroborate ($NEt_4BF_4$; $TEABF_4$), tetrapropylammonium tetrafluoroborate ($NPr_4BF_4$; $TPABF_4$), tetrabutylammonium tetrafluoroborate ($NBu_4BF_4$; $TBABF_4$), and tetrahexylammonium tetrafluoroborate ($NHx_4BF_4$; $THABF_4$) were dried under vacuum at 100 °C for 48 h before being transferred to a $N_2$-filled glovebox. Note that $THABF_4$ was observed to melt at 100 °C in the vacuum oven. Anhydrous acetonitrile (ACN) was purged with $N_2$ for 3 h before taking it into a $N_2$-filled glovebox, where it was further dried by the addition of activated 3 Å molecular sieves. Molecular sieves were activated at 250 °C in a vacuum oven for 12 h prior to transferring into the $N_2$-filled glovebox.

Any unexpected observations and safety hazards are noted below.

## Synthesis

A previously published procedure was used to synthesize $Cu_3(HHTP)_2$.[1] In brief, a solution of $Cu(NO_3)_2 \cdot 3H_2O$ (0.127 g, 0.526 mmol, 1.65 eq.) and aqueous ammonia (35%, 0.883 mL, 50 eq.) in distilled water (2 mL) was prepared. The resulting royal blue solution was added dropwise to a dispersion of HHTP (0.103 g, 0.318 mmol, 1.00 eq.) in distilled water (8.4 mL). The resulting mixture was heated in a furnace oven at 80 °C for 24 h. The dark blue precipitate formed was separated by centrifugation. The precipitate was then washed successively with water (3 × 30 mL), ethanol (3 × 30 mL), and acetone (3 × 30 mL). The precipitate was then filtered by vacuum filtration, and the resulting dark blue powder was dried at 80 °C under dynamic vacuum for 96 h on a Schlenk line before being stored in a $N_2$-filled glovebox until used.

## Materials Characterisation

SEM imaging was performed on a Tescan MIRA3 FEG-SEM. Samples were secured onto stainless steel SEM stubs using adhesive high purity carbon tabs before being sputter-coated with a thin layer (~10 nm) of Pt using a Quorum Technologies Q150T ES Turbo-Pumped Sputter Coater. Imaging was conducted with a beam voltage of 5 kV and working distances of 4 – 5 mm.

Cu content was determined via inductively coupled plasma optical emission spectroscopy using a Thermo Scientific iCAP-7400 ICP spectrometer. C, H and N concentrations were determined via CHN combustion analysis using an Exeter Analytical CE-440, with combustion at 975 °C.

In-house powder XRD data were collected on a Malvern Panalytical Empyrean instrument, equipped with an X'celerator Scientific detector using non-monochromated Cu $K_\alpha$ radiation ($\lambda$ = 1.5418 Å). Samples were placed in a glass sample holder and measured in reflection geometry with sample spinning. The data were collected at room temperature over a $2\theta$ range of 3 – 50 °, with an effective step size of 0.017 ° and a total collection time of 1 h. Simulated XRD patterns were produced using VESTA version 4.6.0. Computational structures used to produce the simulated patterns are available at: https://doi.org/10.5281/zenodo.4694845.

Low-pressure $N_2$ isotherms (adsorption and desorption) were collected using an Anton Parr Autosorb iQ-XR at 77 K. Ex situ degassing (90 °C, 24 h) was performed and isotherms were

collected over 24 – 36 h. Sorption isotherms were evaluated in AsiQwin version 5.21 software. Material BET areas were calculated from isotherms using the BET equation and Rouquerol's consistency criteria implemented in AsiQwin.[2] All pore size distribution fittings were conducted in AsiQwin using $N_2$ at 77 K on carbon (cylindrical pores) quenched solid density functional theory (QSDFT) model with a bin pore width of 0.5 Å.

## Electrode Film Preparation

Freestanding composite $Cu_3(HHTP)_2$ and YP80F films were prepared using existing literature methods.[3] In brief, the electroactive components were ground together in a vial before ethanol (*ca.* 1.5 mL) was added to produce a loose slurry. This was sonicated for 15 min before being added to PTFE dispersion (60 wt. % in water) in a few drops of ethanol. The slurry was stirred by hand for approximately 20 min under ambient conditions. The film was formed upon drying of the slurry and was then kneaded for 20 min to ensure homogeneity before being rolled into a freestanding electrode film using a homemade aluminium rolling pin. The film was dried at 100 °C under dynamic vacuum for at least 48 h to remove remaining ethanol. The masses of components were calculated so that the final $Cu_3(HHTP)_2$ composite films had a composition of 85 wt. % $Cu_3(HHTP)_2$, 10 wt. % acetylene black (measured BET area = 62 $m^2$ $g^{-1}$), and 5 wt. % PTFE. YP80F composite films were made with 85 wt. % YP80F, 10 wt. % acetylene black, and 5 wt. % PTFE. All films had a thickness of *ca*. 250 μm.

## Electrochemical Measurements

Three-electrode cells were prepared in Swagelok PFA-820-3 union tube fittings with homemade stainless-steel plugs as current collectors. $Cu_3(HHTP)_2$ composite electrodes with areal mass loadings ranging between 10 – 14 mg $cm^{-2}$ were used as working electrodes. Overcapacitive YP80F activated carbon film electrodes with areal mass loadings of 35 – 40 mg $cm^{-2}$ were used as counter electrodes. Both the working and counter electrodes were cut with diameters of ¼ ", giving a typical mass ratio between the working and counter electrode of approximately 1:3.2 on average. Ag wire was used as a pseudo-reference electrode. 1 M solutions of $TEABF_4$, $TPABF_4$, $TBABF_4$, and $THABF_4$ in anhydrous acetonitrile were used as electrolytes. The amount of electrolyte added was kept constant between cells (750 μL). Whatman glass microfiber filter (GF/A) was used as a separator, and two separators were added to each cell. The cells were hermetically sealed by hand and removed from the glovebox for testing. Under these conditions, the midpoint potential of the ferrocene–ferricenium ($Fc/Fc^+$) redox couple was measured at 0.564 ± 0.002 V *vs* Ag. All potentials discussed for the three-electrode cell are referenced to Ag.

Symmetric two-electrode supercapacitor cells were assembled as coin cells in Cambridge Energy Solutions CR2032 SS316 coin cell cases. Electrodes were cut from freestanding composite $Cu_3(HHTP)_2$ films with areal mass loadings ranging between 13.4 – 16.0 mg $cm^{-2}$ for cells made with $Cu_3(HHTP)_2$ Sample 1, and between 9.9 – 11.8 mg $cm^{-2}$ for cells made with $Cu_3(HHTP)_2$ Sample 2. 1 M solutions of $TEABF_4$, $TPABF_4$, $TBABF_4$, and $THABF_4$ in anhydrous acetonitrile were used as electrolytes. The amount of electrolyte added was kept constant between cells (200 μL). Whatman glass microfiber filter (GF/A) was used as a separator, and two separators were added to each cell. Each coin cell contained two SS316 separator disks and one SS316 spring to ensure sufficient and consistent pressure between cells. The coin cells were sealed in the glovebox using a Compact Hydraulic Coin Cell Crimper (Cambridge Energy Solutions) before being removed for testing.

All electrochemical measurements on two- and three-electrode cells were carried out using Biologic VSP-3e and SP-150 potentiostats. All experimental capacity and capacitance values

for $Cu_3(HHTP)_2$ were calculated after removing the contributions of acetylene black and PTFE that are also present in the electrodes. For three-electrode experiments, all reported specific capacity and specific capacitance values were normalised by the mass of $Cu_3(HHTP)_2$ in the working electrode. For two-electrode experiments, specific capacity values are normalised by the total mass of $Cu_3(HHTP)_2$ in the two-electrode cell assembly, while specific capacitance values are normalised by the average mass of $Cu_3(HHTP)_2$ in a single electrode (i.e., a pseudo single electrode measurement independent of device architecture).

EQCM measurements were performed with an AT-cut Au-coated quartz crystal (AWSensors) with an oscillating frequency of 9 MHz. A slurry containing 85 wt. % $Cu_3(HHTP)_2$ powder, 10 wt. % acetylene black, and 5 wt. % polyvinylidene fluoride (PVDF; Arkema) binder in N-Methyl-2-pyrrolidone was spray-coated onto the Au-coated surface of the quartz crystal. The sample-coated quartz crystal was dried at 80 °C under dynamic vacuum for 24 h to remove remaining PVDF, and then used as the working electrode in EQCM cells. Platinum wire was used as the counter electrode, and Ag wire was used as a pseudo-reference electrode. 1 M solutions of $TEABF_4$ and $THABF_4$ in anhydrous acetonitrile were used as electrolytes. All EQCM cells were assembled in a $N_2$-filled glovebox. EQCM electrochemical measurements were carried out using a Metrohm Autolab electrochemical workstation and a Maxtek RQCM system in tandem to allow for simultaneous recording of frequency and electrochemistry data.

**SI Table S1: Predicted & Experimental Elemental Analysis Results from $Cu_3(HHTP)_2$**

| Element | Predicted / wt% | Sample 1 / wt% | Sample 2 / wt% |
|---|---|---|---|
| Cu | 23.1 | 24.2 | 22.1 |
| C | 52.3 | 46.1 | 48.2 |
| H | 1.5 | 2.4 | 2.1 |
| N | 0 | 3.3 | 3.2 |

(a)

(b)

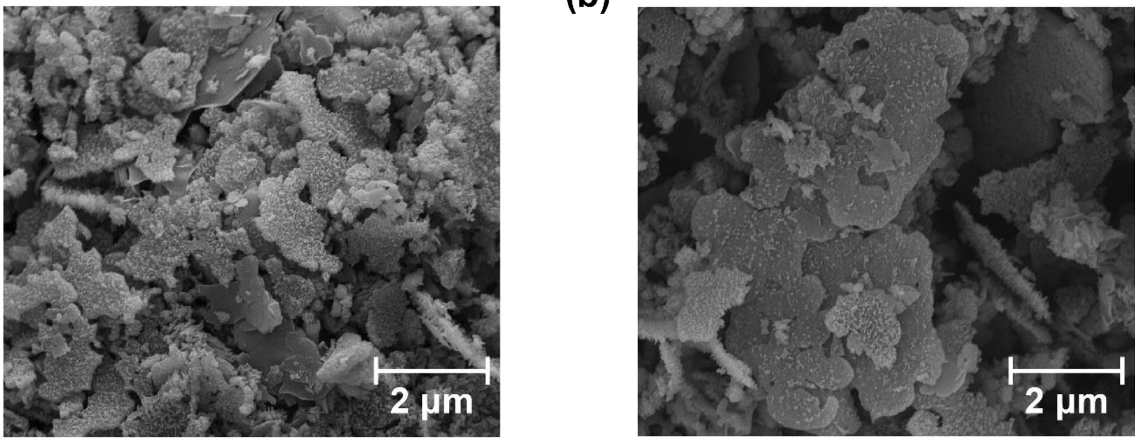


**SI Figure S1**: Scanning electron microscopy (SEM) images from two samples of $Cu_3(HHTP)_2$ synthesised for this work: (a) Sample 1, and (b) Sample 2. These show that the samples used in this study have a flake-like crystallite morphology. Two samples of $Cu_3(HHTP)_2$ (Sample 1 and Sample 2) were synthesised for this work and tested throughout to ensure that the results seen were consistent between different sample batches of the MOF. The data from different samples are labelled accordingly.

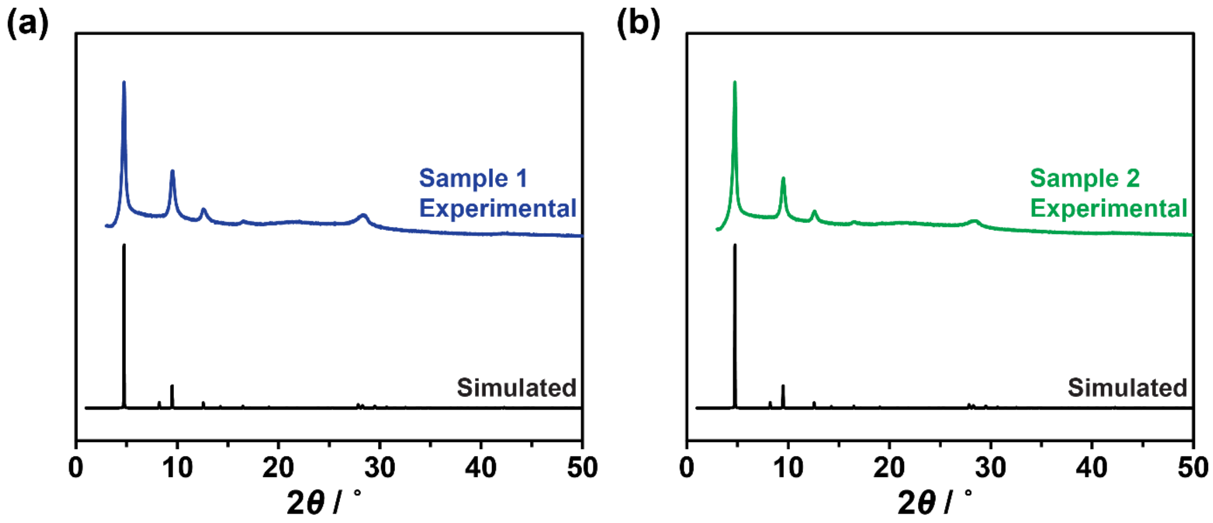


**SI Figure S2**: Experimental powder XRD patterns from two powder samples of $Cu_3(HHTP)_2$ synthesised in this work (labelled Sample 1 and Sample 2, respectively, throughout) compared to the simulated XRD pattern for $Cu_3(HHTP)_2$ with an eclipsed stacking sequence. This data confirms the identity and high crystallinity of the as-synthesised $Cu_3(HHTP)_2$ used in this work.

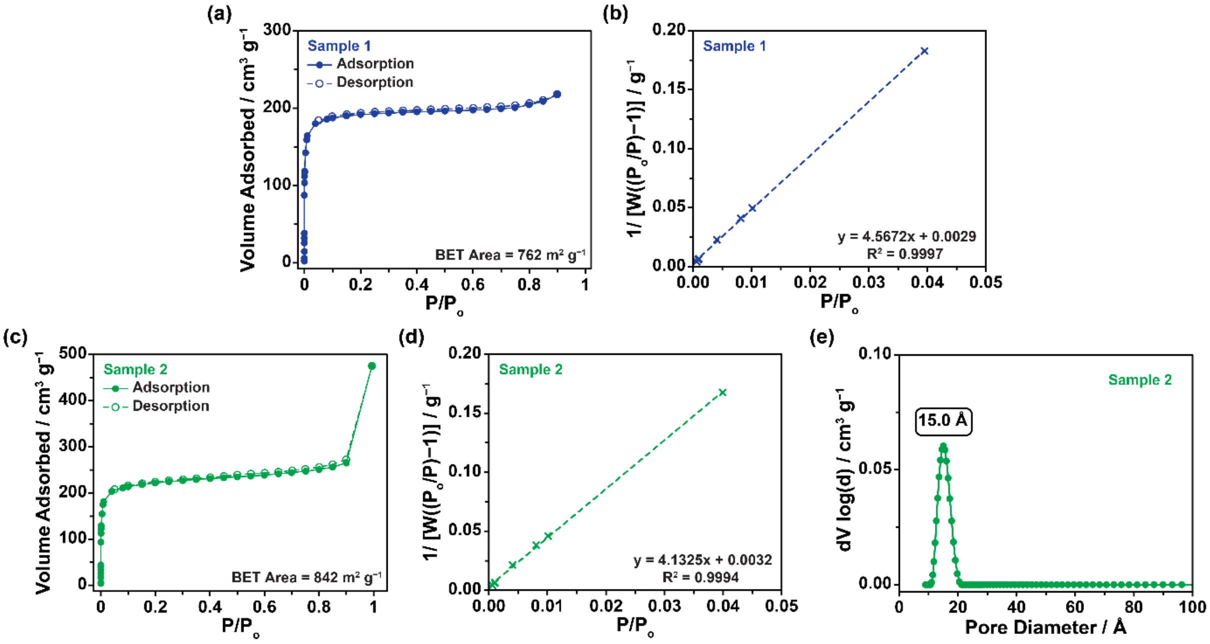


**SI Figure S3**: $N_2$ 77 K gas sorption analysis of two powder samples of $Cu_3(HHTP)_2$ synthesised in this work (blue data from Sample 1; green data from Sample 2). (a) 77 K $N_2$ sorption isotherm of Sample 1 along with (b) the corresponding plot used to calculate the BET surface area of the sample. The pore size distribution (PSD) of this sample is shown in Figure 1 in the Main Text. (c) 77 K $N_2$ sorption isotherm of Sample 2 along with (d) the corresponding plot used to calculate the BET surface area of the sample, and (e) the pore size distribution of the sample calculated from the $N_2$ sorption isotherm using a $N_2$ at 77 K on carbon (cylindrical pores) quenched solid density functional theory (QSDFT) model. For sorption isotherms, adsorption is shown with blocked lines and filled circles, while desorption is shown with dashed lines and unfilled circles. This data confirms the high porosity of the as-synthesised $Cu_3(HHTP)_2$ used in this work.

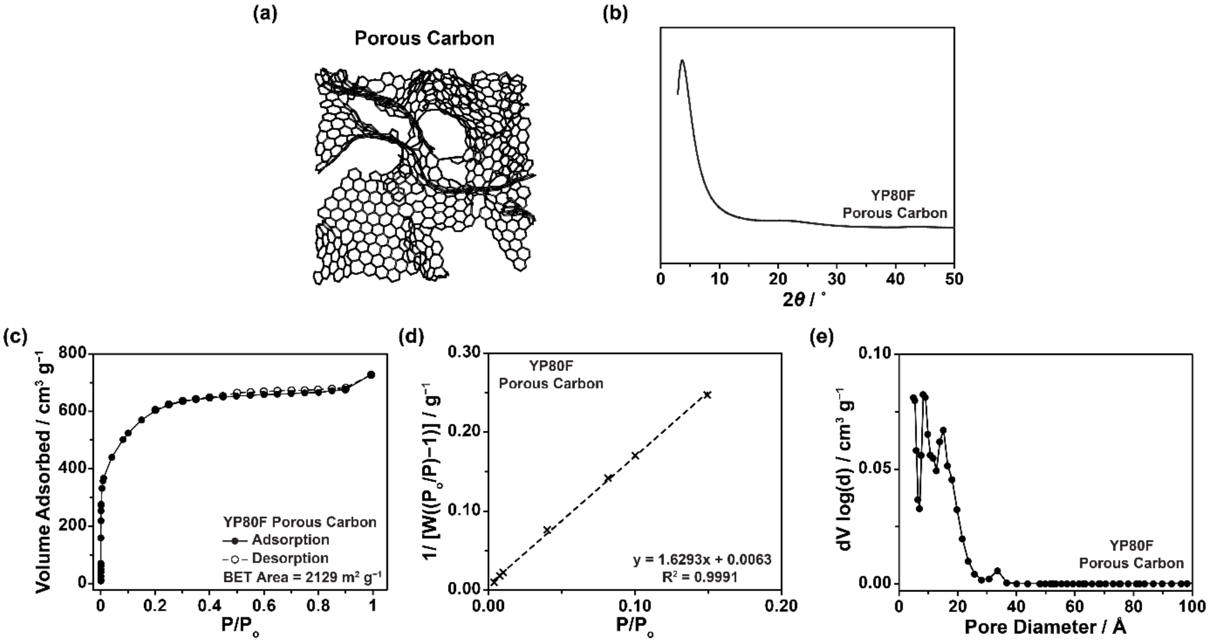


**SI Figure S4**: Characterisation data from a common porous carbon material, YP80F. (a) A schematic of the amorphous carbon structure, showing the disordered pore network of this material. (b) XRD pattern of YP80F powder illustrating the lack of crystallinity and long-range order in this material, in contrast to $Cu_3(HHTP)_2$. (c) 77 K $N_2$ sorption isotherm of YP80F powder along with (d) the corresponding plot used to calculate the BET surface area of the sample, and (e) the pore size distribution of the sample calculated from the $N_2$ sorption isotherm using a $N_2$ at 77 K on carbon (slit pores) quenched solid density functional theory (QSDFT) model. This shows that, while porous carbons typically have greater total porosities than layered MOFs, they have significantly more disordered pore structures. For the sorption isotherm, adsorption is shown with blocked lines and filled circles, while desorption is shown with dashed lines and unfilled circles.

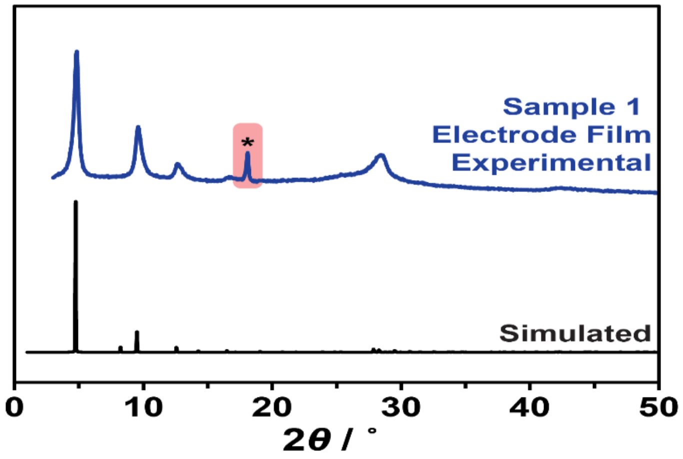


**SI Figure S5**: Experimental XRD pattern from a $Cu_3(HHTP)_2$ composite electrode film produced in this work with Sample 1. This is compared to the simulated XRD pattern for $Cu_3(HHTP)_2$ with an eclipsed stacking sequence. This confirms that the crystallinity of the MOF is maintained upon electrode film formation. The peak highlighted in red is the (100) peak from crystalline PTFE present in the electrode film.[9]

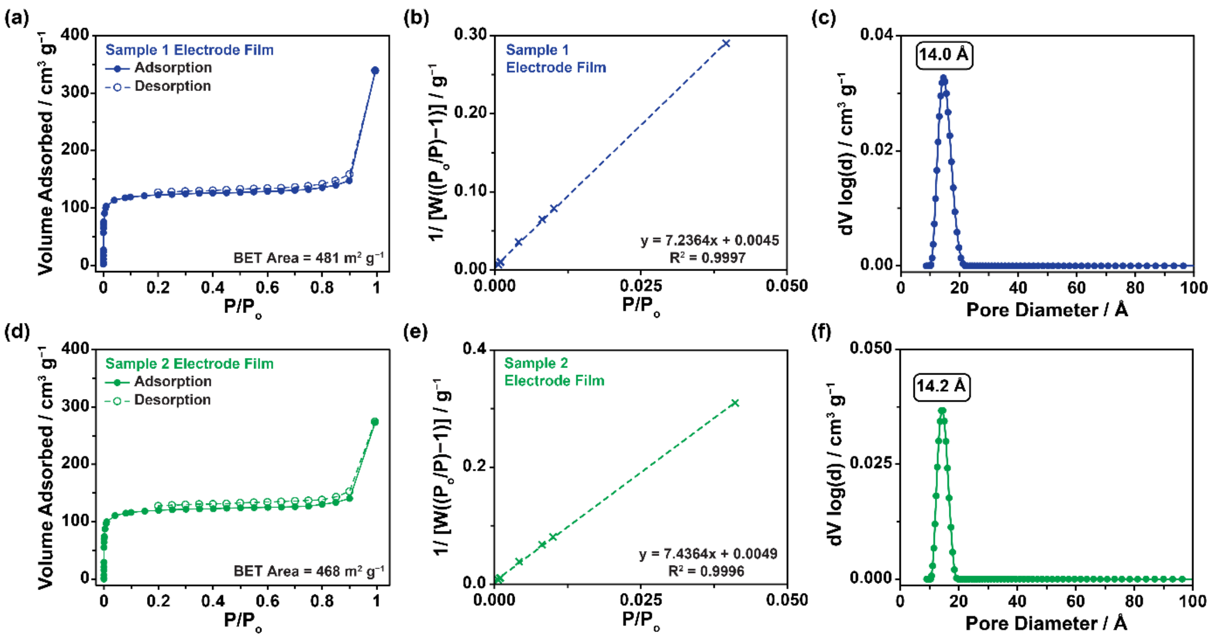


**SI Figure S6**: $N_2$ 77 K gas sorption analysis of two $Cu_3(HHTP)_2$ electrode films produced in this work from two different MOF samples (Sample 1 and Sample 2). The gas sorption analysis of the powder samples is presented in Figure 1 of the Main Text, and in SI Figure S3. (a) 77 K $N_2$ sorption isotherm of Sample 1 Electrode Film along with (b) the corresponding plot used to calculate the BET surface area of the sample, and (c) the pore size distribution of the sample calculated from the $N_2$ sorption isotherm using a $N_2$ at 77 K on carbon (cylindrical pores) quenched solid density functional theory (QSDFT) model. (d) 77 K $N_2$ sorption isotherm of Sample 2 Electrode Film along with (e) the corresponding plot used to calculate the BET surface area of the sample, and (f) the pore size distribution of the sample calculated from the $N_2$ sorption isotherm using a $N_2$ at 77 K on carbon (cylindrical pores) quenched solid density functional theory (QSDFT) model. For sorption isotherms, adsorption is shown with blocked lines and filled circles, and desorption is shown with dashed lines and unfilled circles. This data illustrates the decrease in BET surface area of $Cu_3(HHTP)_2$ that occurs during electrode film preparation.

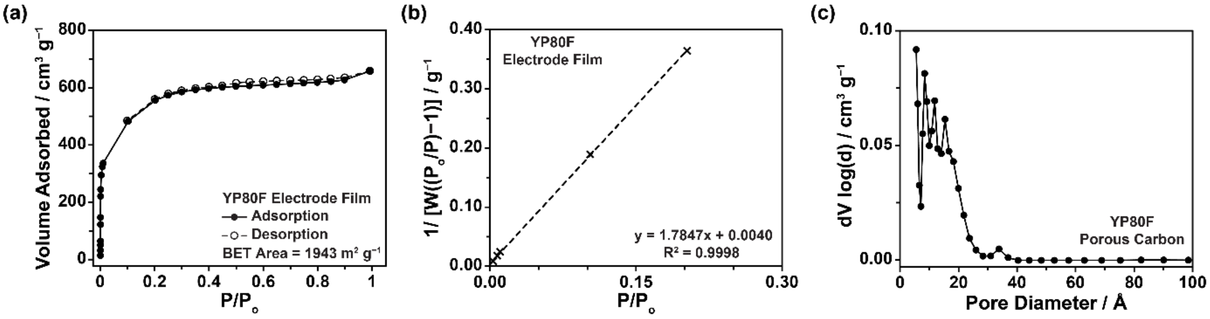


**SI Figure S7**: $N_2$ 77 K gas sorption analysis of a YP80F electrode film produced in this work. The gas sorption analysis of YP80F powder is presented in SI Figure S4. (a) 77 K $N_2$ sorption isotherm of YP80F Electrode Film along with (b) the corresponding plot used to calculate the BET surface area of the sample, and (c) the pore size distribution of the sample calculated from the $N_2$ sorption isotherm using a $N_2$ at 77 K on carbon (slit pores) quenched solid density functional theory (QSDFT) model. For the sorption isotherm, adsorption is shown with blocked lines and filled circles, and desorption is shown with dashed lines and unfilled circles. This data shows that the percentage decrease in BET surface area of YP80F that occurs during film preparation is significantly lower than that of $Cu_3(HHTP)_2$.

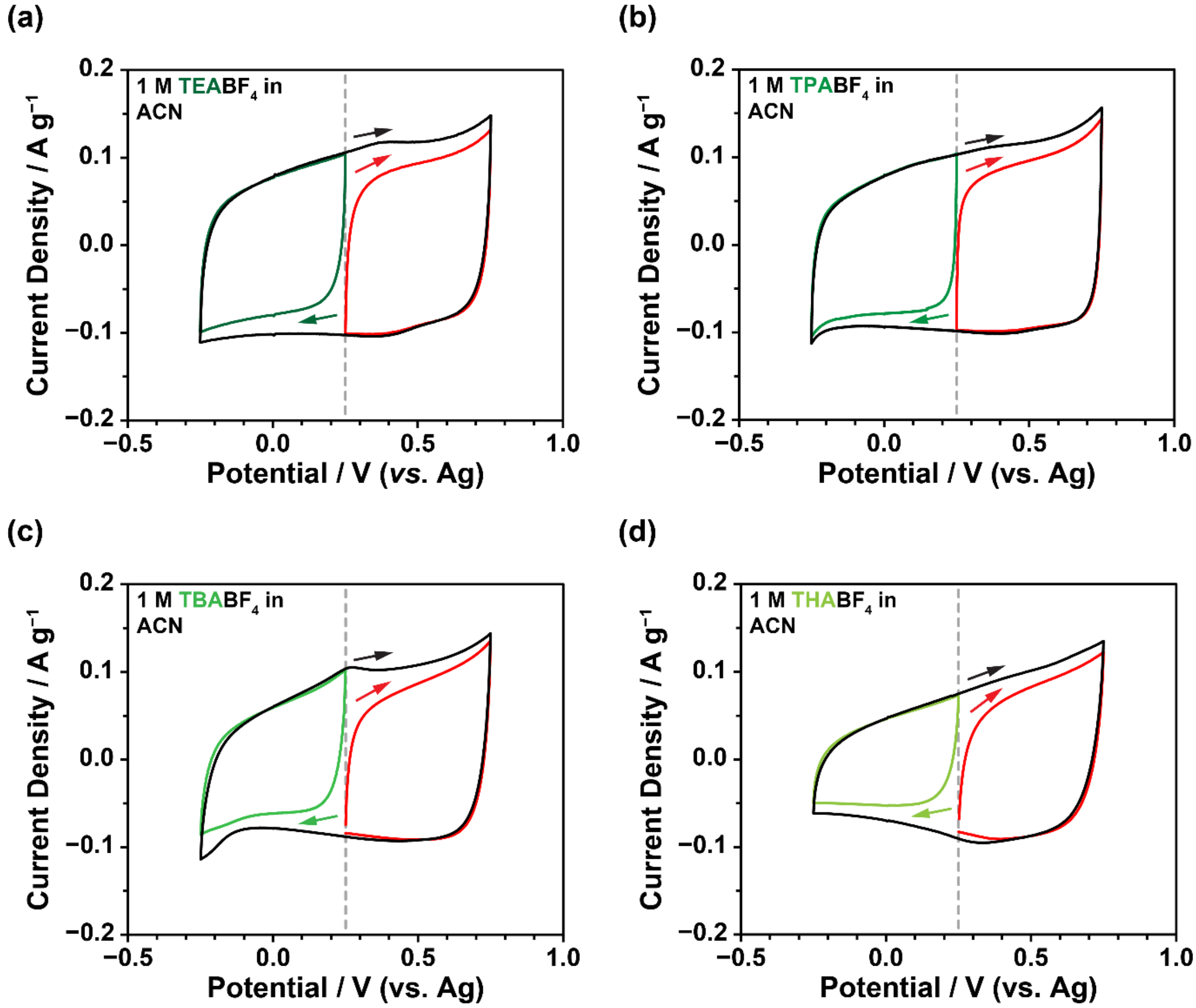


**SI Figure S8**: Repeat cyclic voltammetry (CV) data obtained at a scan rate of 1 mV $s^{-1}$ from three-electrode cells assembled with $Cu_3(HHTP)_2$ (Sample 2) working electrodes, YP80F oversized counter electrodes, and Ag pseudo-reference electrodes, with 1 M solutions of (a) $TEABF_4$, (b) $TPABF_4$, (c) $TBABF_4$, and (d) $THABF_4$ in acetonitrile as electrolytes. The open circuit potential (OCP) is indicated for each CV by the grey dashed line. Data was acquired by scanning to +0.5 V *vs*. OCP (red), −0.5 V *vs*. OCP (blue), and across the full potential window (black). The direction of scanning is indicated by the arrow in each case. This data was acquired on a different sample of $Cu_3(HHTP)_2$ than the CV data presented in Figure 2 of the Main Text, and confirms the reproducibility of the results. Specific capacity values from these cells, calculated from galvanostatic charge-discharge (GCD) profiles, are presented as part of Figure 2f in the Main Text, as well as in SI Table S2 and Figure S9.

**SI Table S2: Specific Capacity and Capacitance Values at 0.05 A $g^{-1}$ Calculated from Galvanostatic Charge-Discharge Experiments on 3-Electrode Cells**

| Cell | Specific Capacity / C $g^{-1}$ | | Specific Capacitance / F $g^{-1}$ | |
|---|---|---|---|---|
| | +0.5 V *vs*. OCP | −0.5 V *vs*. OCP | +0.5 V *vs*. OCP | −0.5 V *vs*. OCP |
| TEA Cell 1 | 51.0 | 44.0 | 102.0 | 88.0 |
| TPA Cell 1 | 45.8 | 42.4 | 91.6 | 84.8 |
| TBA Cell 1 | 39.7 | 28.7 | 79.4 | 57.4 |
| THA Cell 1 | 39.8 | 18.1 | 79.7 | 36.1 |
| TEA Cell 2 | 49.1 | 40.2 | 98.3 | 80.4 |
| TPA Cell 2 | 48.0 | 38.4 | 96.0 | 76.8 |
| TBA Cell 2 | 45.5 | 33.6 | 91.0 | 67.1 |
| THA Cell 2 | 43.3 | 20.8 | 86.6 | 41.6 |

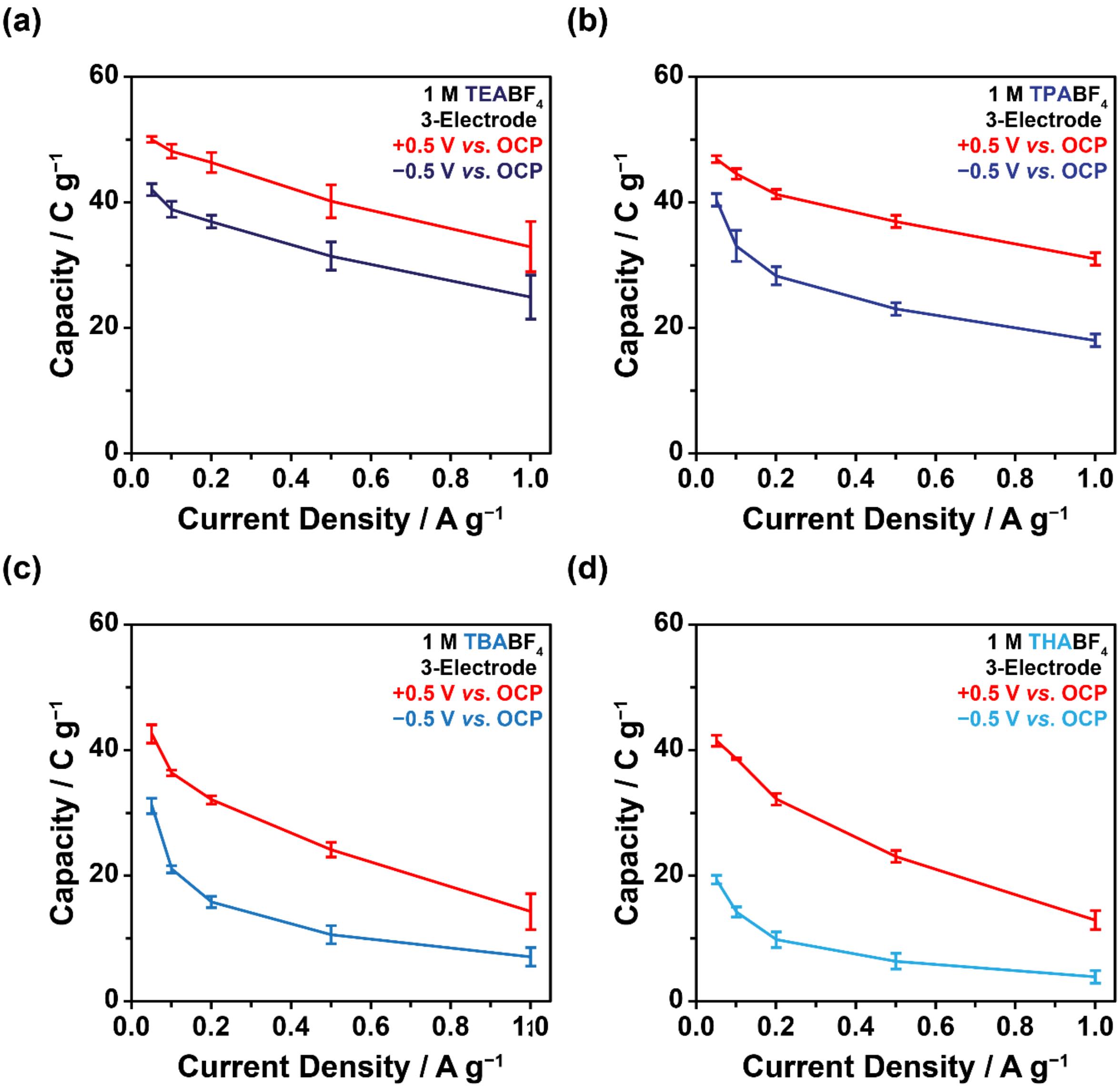


**SI Figure S9**: Capacity *vs*. current density plots from three-electrode cells assembled with $Cu_3(HHTP)_2$ working electrodes, YP80F oversized counter electrodes, and Ag pseudo-reference electrodes, with 1 M solutions of (a) $TEABF_4$, (b) $TPABF_4$, (c) $TBABF_4$, and (d) $THABF_4$ in acetonitrile as electrolytes. Data was acquired by scanning to both +0.5 V *vs*. OCP (red) and −0.5 V *vs*. OCP (blue), showing differences in the rate capability for each system upon both positive and negative charging. This highlights the lower power performance for systems with larger $TAA^+$ cations, suggesting lower ion mobilities for these electrolyte species in the pores of $Cu_3(HHTP)_2$. This is summarised below in SI Table S3. Data was obtained using $Cu_3(HHTP)_2$ Samples 1 and 2. All specific capacity values were calculated from GCD experiments using only the mass of electroactive material (i.e. $Cu_3(HHTP)_2$) in the working electrode.

**SI Table S3: Rate Capability Values for Charging Between 0.05 – 1 A $g^{-1}$ Calculated from Galvanostatic Charge-Discharge Experiments on 3-Electrode Cells**

| Cation | Rate Capability* / % | |
|---|---|---|
| | +0.5 V *vs.* OCP | −0.5 V *vs.* OCP |
| TEA | 65.9 | 59.4 |
| TPA | 66.1 | 44.6 |
| TBA | 33.5 | 22.6 |
| THA | 31.5 | 20.6 |

* In this work, the rate capability is defined as the percentage capacitance retention at a current density of 1 A $g^{-1}$ compared to the capacitance value at 0.05 A $g^{-1}$ (the lowest current density used in this work).

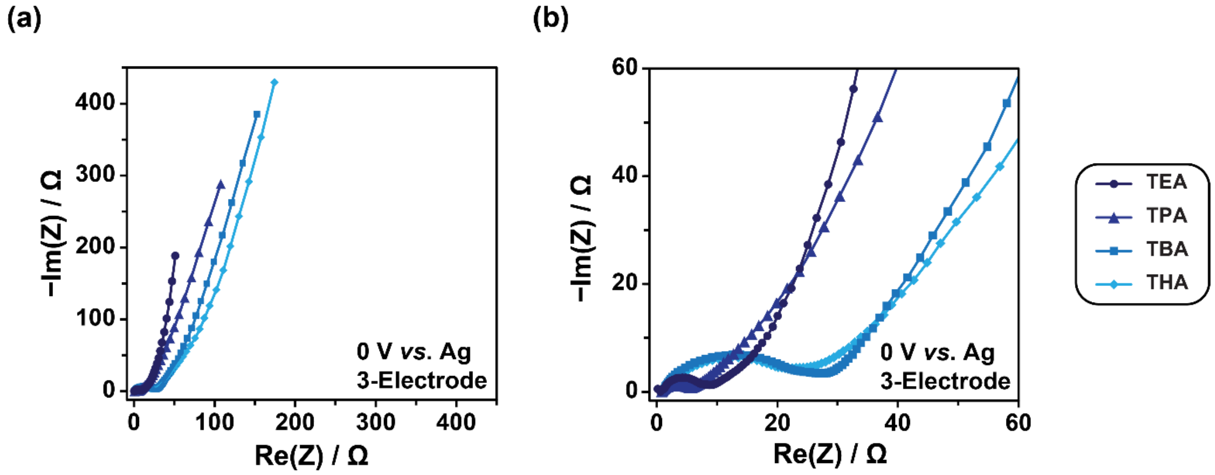


**SI Figure S10**: Nyquist plots from electrochemical impedance spectroscopy (EIS) experiments performed at a constant bias potential of 0 V *vs.* Ag on three-electrode cells assembled with $Cu_3(HHTP)_2$ working electrodes (Sample 1), YP80F oversized counter electrodes, and Ag pseudo-reference electrodes and 1 M solutions of $TEABF_4$, $TPABF_4$, $TBABF_4$, and $THABF_4$ in acetonitrile as electrolytes. Impedance data is for the $Cu_3(HHTP)_2$ working electrode. (a) shows the full data set to highlight differences in the low frequency responses between the different electrolytes. (b) shows a zoomed view to highlight the high and intermediate frequency domains. The EIS data shows differences in the ion mobility between the different electrolytes from the different slopes of low frequency response.

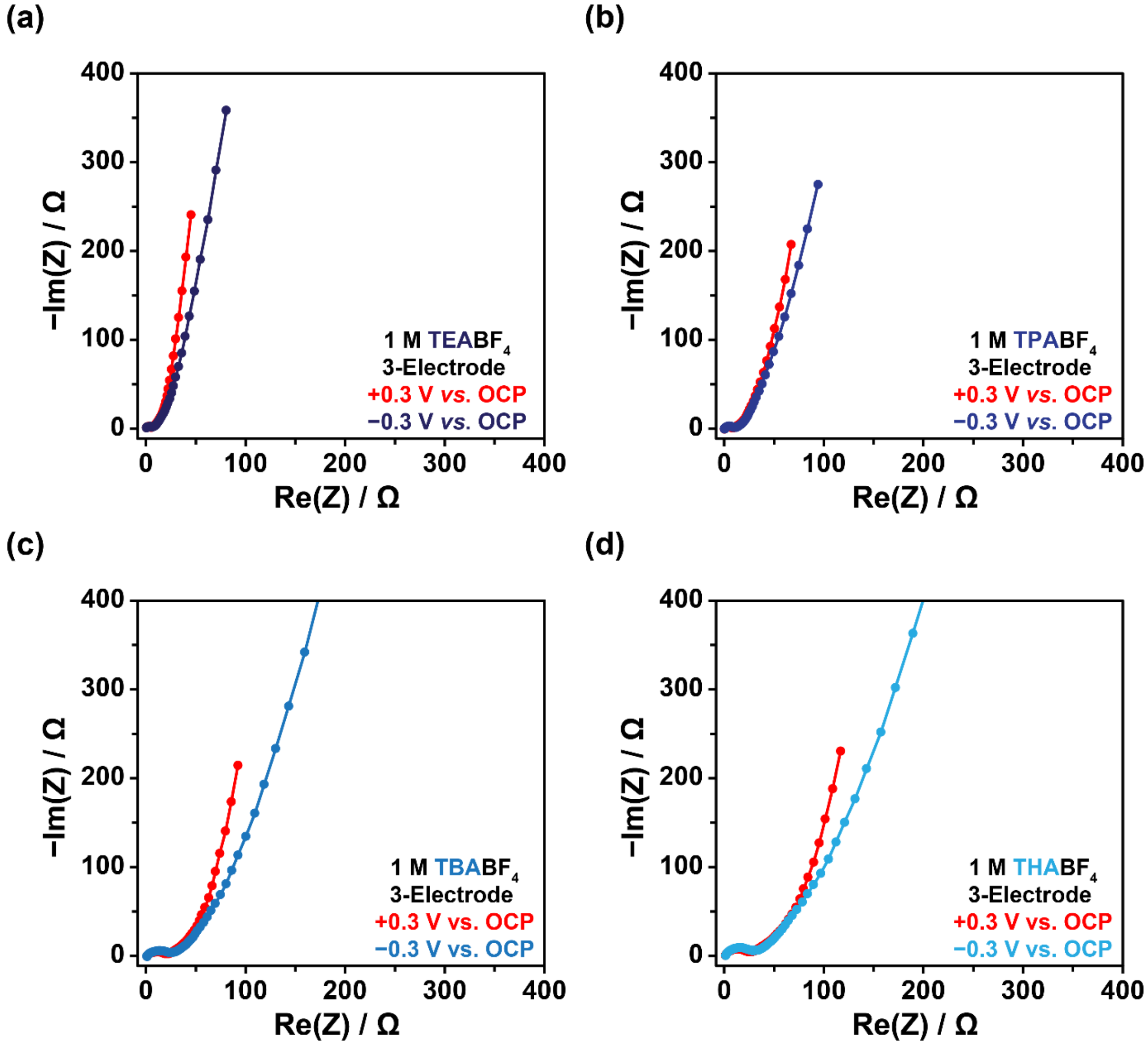


**SI Figure S11**: Nyquist plots from electrochemical impedance spectroscopy (EIS) experiments performed at potentials of +0.3 V *vs.* OCP (red) and −0.5 V *vs.* OCP (blue) on three-electrode cells assembled with $Cu_3(HHTP)_2$ working electrodes (Sample 1), YP80F oversized counter electrodes, and Ag pseudo-reference electrodes and 1 M solutions of (a) $TEABF_4$, (b) $TPABF_4$, (c) $TBABF_4$, and (d) $THABF_4$ in acetonitrile as electrolytes. Impedance data is for the $Cu_3(HHTP)_2$ working electrode. This data shows the differences in ion mobility (slope of low frequency response) between positive and negative charging for each of the different electrolytes, and illustrates this difference is greater for electrolytes with larger cations, which show lower ion mobility upon negative charging.

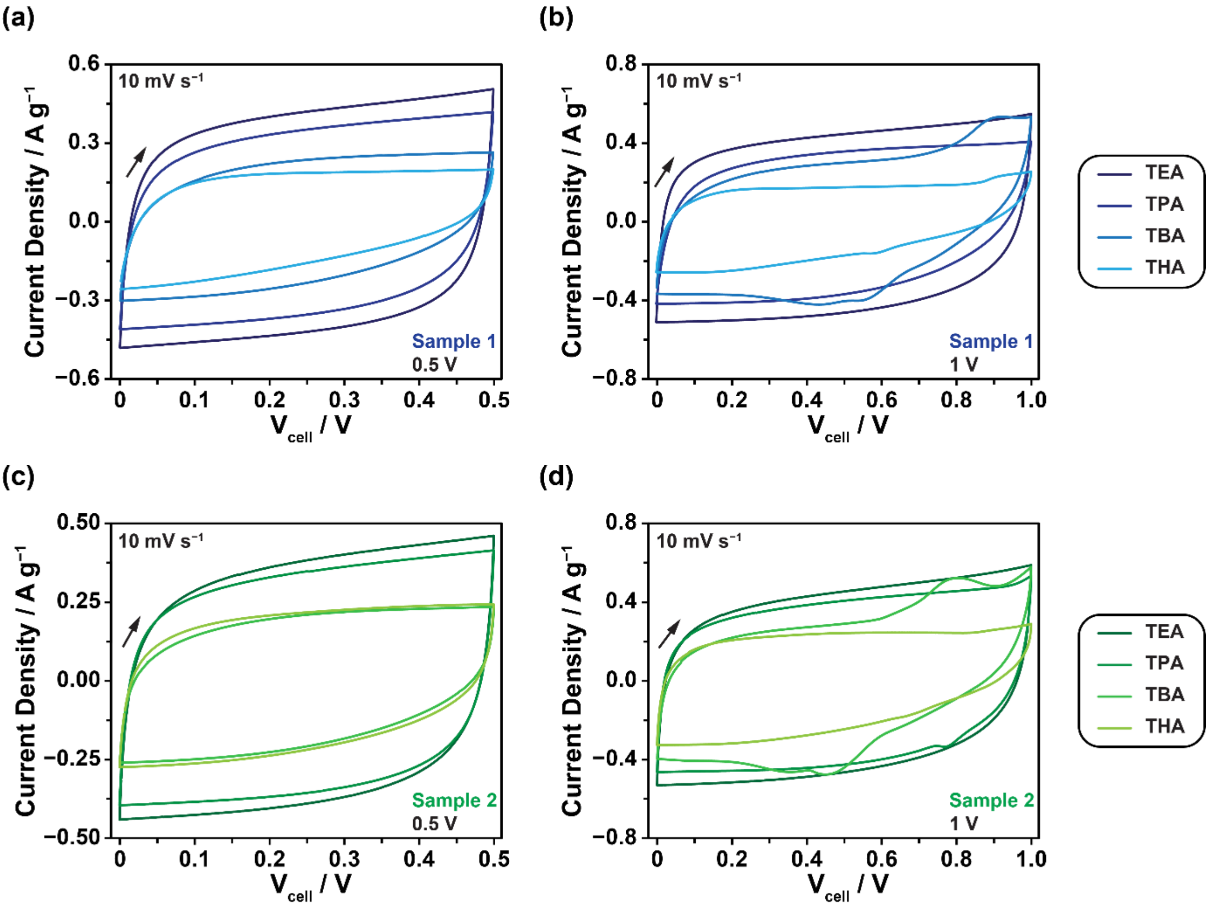


**SI Figure S12**: Cyclic voltammetry (CV) data obtained at a scan rate of 10 mV $s^{-1}$ from symmetric two-electrode cells assembled with $Cu_3(HHTP)_2$ composite film electrodes and 1 M solutions of $TEABF_4$, $TPABF_4$, $TBABF_4$, and $THABF_4$ in acetonitrile as electrolytes. Data from two independent cells, each made using different samples of $Cu_3(HHTP)_2$, is shown (blue data from Sample 1; green data from Sample 2). CV data is shown limited to two different final cell voltages. (a) and (c) show CVs limited to 0.5 V, within the double-layer stability window for all the electrolytes used. This illustrates the difference in charge storage between the different electrolytes, with smaller $TAA^+$ cations giving higher charge storage. (b) and (d) show CVs limited to 1 V, showing the differences in the cell voltage at which faradaic activity occurs in the different systems. This is a consequence of the greater asymmetry in charging with larger $TAA^+$ cations.

(a)

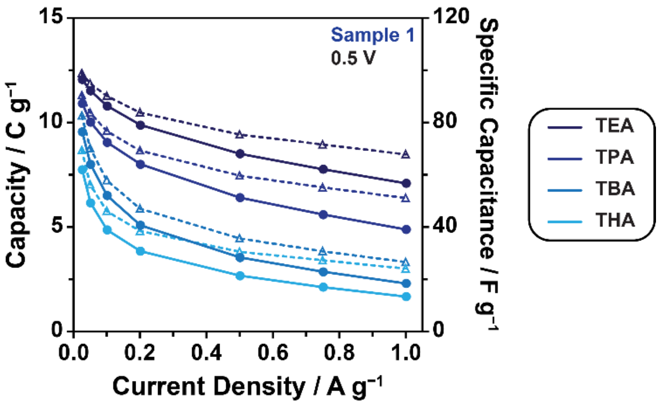


(b)

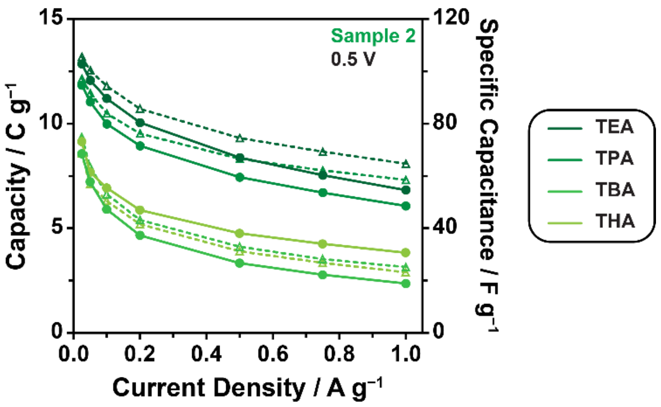


**SI Figure S13**: Capacity and specific capacitance values obtained from galvanostatic charge-discharge (GCD) experiments performed at a range of different current densities on symmetric two-electrode cells assembled with $Cu_3(HHTP)_2$ composite film electrodes and 1 M solutions of $TEABF_4$, $TPABF_4$, $TBABF_4$, and $THABF_4$ in acetonitrile as electrolytes. Data from two independent cells, each made using different samples of $Cu_3(HHTP)_2$, is shown (blue data from Sample 1; green data from Sample 2). Capacity values are shown with filled circles and bold lines, and have been calculated for the full cell by dividing the calculated capacity by the total mass of active electrode material in the two-electrode cell assembly. Specific capacitance values are shown with unfilled triangles and dashed lines, and have been calculated for the active electrode material in a single electrode (i.e., a pseudo single electrode measurement independent of device architecture).

**SI Table S4: Capacity and Specific Capacitance Values at 0.05 A $g^{-1}$ Calculated from Galvanostatic Charge-Discharge Experiments on 2-Electrode Cells at 0.5 V**

| Cell | *Capacity / C $g^{-1}$ | **Specific Capacitance / F $g^{-1}$ |
|---|---|---|
| TEA Cell 1 | 12.1 | 98.7 |
| TPA Cell 1 | 10.9 | 90.2 |
| TBA Cell 1 | 9.6 | 82.5 |
| THA Cell 1 | 7.7 | 69.3 |
| TEA Cell 2 | 12.9 | 105.5 |
| TPA Cell 2 | 11.8 | 97.3 |
| TBA Cell 2 | 8.6 | 74.9 |
| THA Cell 2 | 8.6 | 73.0 |

*Capacity have been calculated for the full cell by dividing the calculated capacity by the total mass of active electrode material in the two-electrode cell assembly.

**Specific capacitance values have been calculated for the active electrode material in a single electrode (i.e., a pseudo single electrode measurement independent of device architecture).

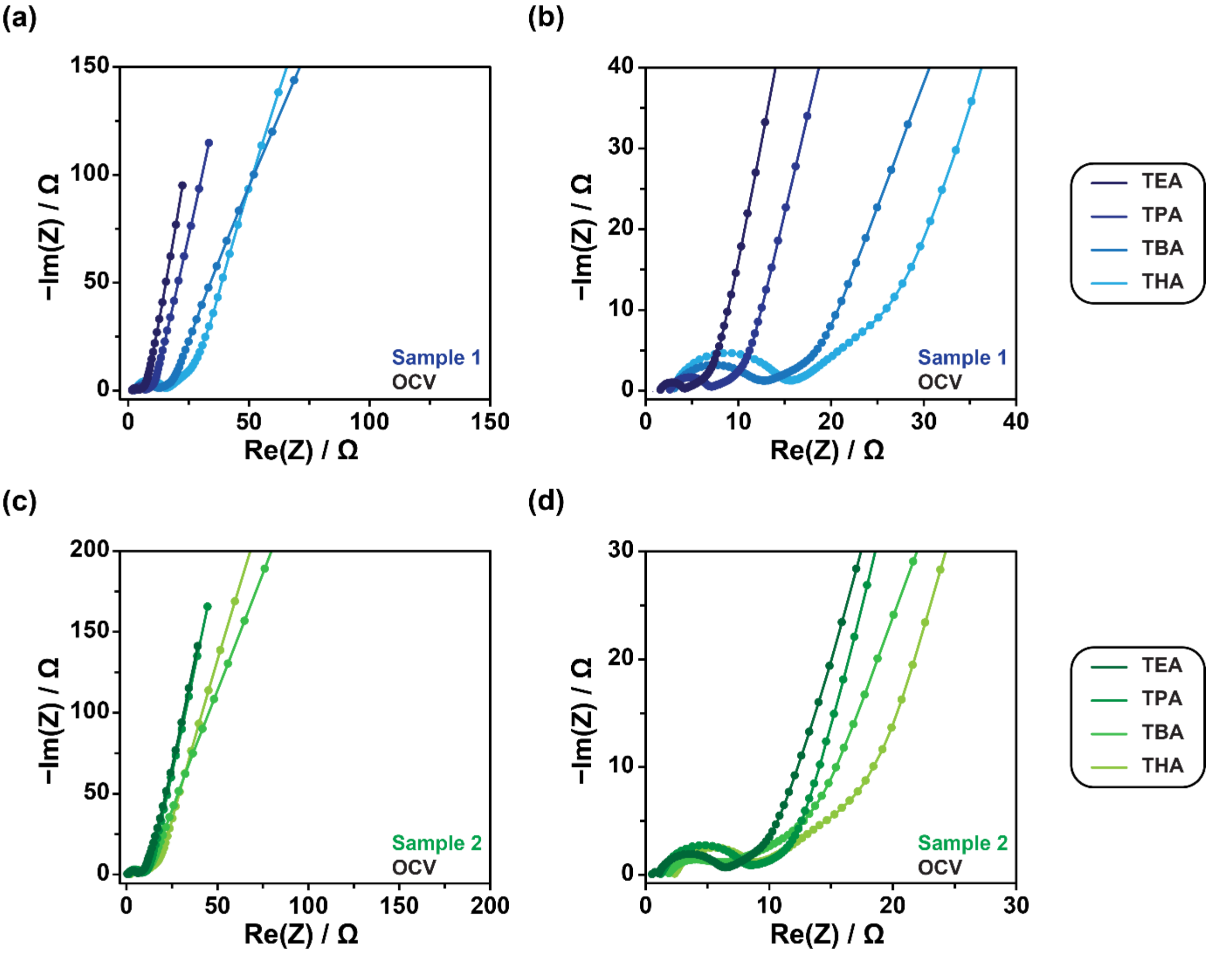


**SI Figure S14**: Nyquist plots from electrochemical impedance spectroscopy (EIS) experiments performed at the open circuit voltage (OCV) on symmetric two-electrode cells assembled with $Cu_3(HHTP)_2$ composite film electrodes and 1 M solutions of $TEABF_4$, $TPABF_4$, $TBABF_4$, and $THABF_4$ in acetonitrile as electrolytes. Data from two independent cells, each made using different samples of $Cu_3(HHTP)_2$, is shown (blue data from Sample 1; green data from Sample 2). (a) and (c) show the full data set to highlight differences in the low frequency responses between the different electrolytes. (b) and (d) show a zoomed view to highlight the high and intermediate frequency domains. The EIS data shows differences in the ion mobility (slope of low frequency response) between the different electrolytes .

**SI Table S5: Comparison of Areal Capacitance Values Obtained for $Cu_3(HHTP)_2$ with 1 M $TEABF_4$ in Acetonitrile Electrolyte from Three-Electrode Experiments in This Work with Simulated Capacitance Values Obtained from Quantum-Mechanics/Molecular-Mechanics (QM/MM) Simulations Performed with Different Charging Mechanisms**[4]

| QM/MM Simulated Areal Capacitance Values[4] | | | |
|---|---|---|---|
| *X* Value | Charging Mechanism | Negative Charging / μF $cm^{-2}$ | Positive Charging / μF $cm^{-2}$ |
| 1.0 | Counterion Adsorption | 18 | 8 |
| 0.5 | | 12 | 12 |
| 0.0 | Ion Exchange | 8 | 12 |
| −0.5 | | 7 | 11 |
| −1.0 | Co-ion Desorption | 6 | 24 |
| Experimental Values from This Work | | | |
| From three-electrode measurements | | 15.1 ± 1.3 | 17.9 ± 1.0 |

**Areal capacitance values have been calculated for the active electrode material in a single electrode (i.e., a pseudo single electrode measurement independent of device architecture), consistent with the method used in the QM/MM simulations.

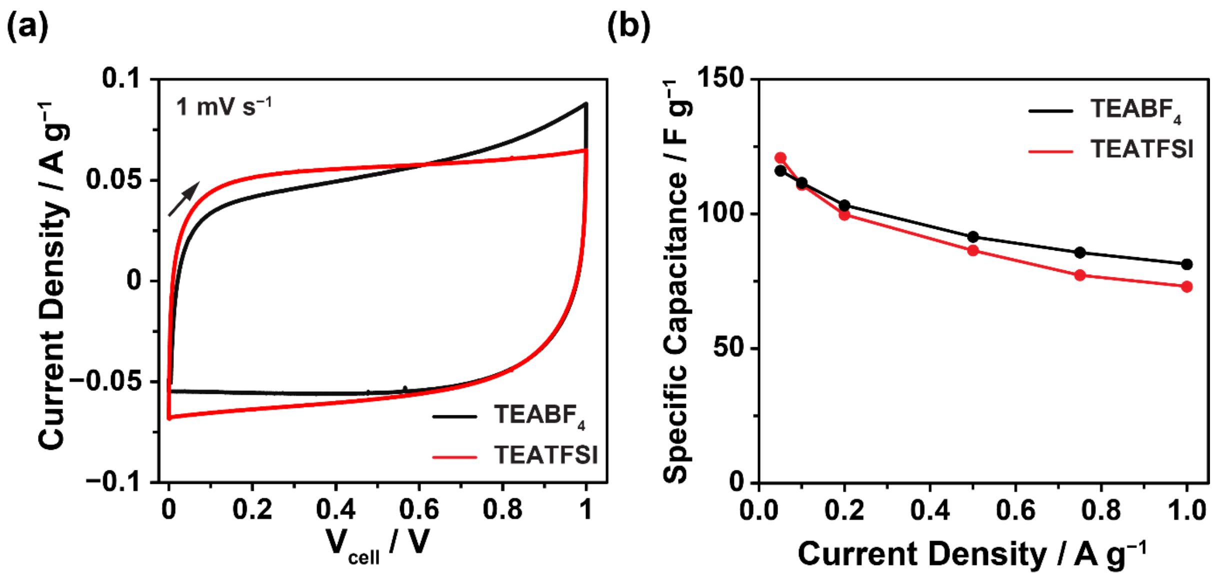


**SI Figure S15**: (a) CVs obtained at a scan rate of 1 mV $s^{-1}$ from two-electrode symmetric supercapacitors assembled with $Cu_3(HHTP)_2$ electrodes and 1 M solutions of tetraethylammonium tetrafluoroborate ($TEABF_4$; black) and tetraethylammonium bis(trifluoromethanesulfonyl)imide (TEATFSI; red) in acetonitrile electrolytes. The direction of scanning is indicated by the arrow. (b) Specific capacitance vs. current density plots from the same two-electrode cells showing minimal differences in the specific capacitance and rate capability for between the two different electrolytes. These experiments indicate that there is no effect of the anion on the charge storage performance of $Cu_3(HHTP)_2$.

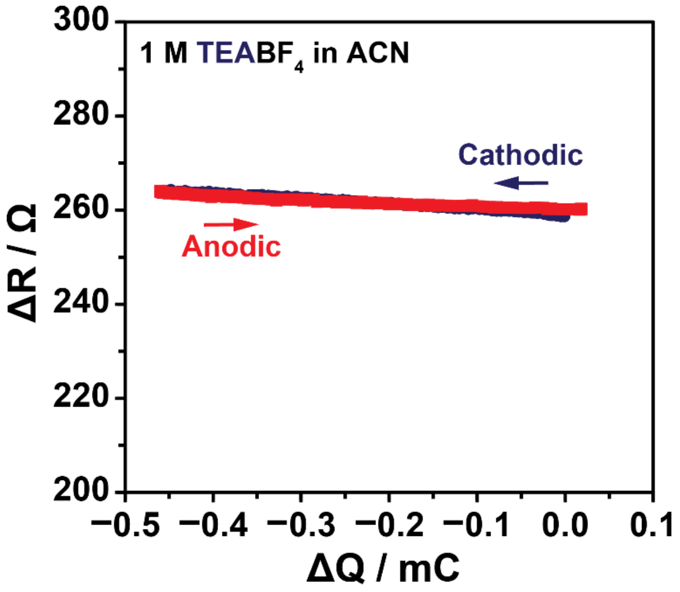


**SI Figure S16**: Motional resistance (ΔR) *vs*. charge (ΔQ) plot from electrochemical quartz crystal microbalance cell assembled with a $Cu_3(HHTP)_2$-coated quartz working electrode, platinum wire counter electrode, a Ag pseudo-reference electrode, and 1 M $TEABF_4$ in acetonitrile as the electrolyte. This shows that there was negligible change in motional resistance during CV cycling, suggesting a homogenous and rigid coating of $Cu_3(HHTP)_2$ on the surface of the quartz crystal that did not detach from the crystal during electrochemical testing.

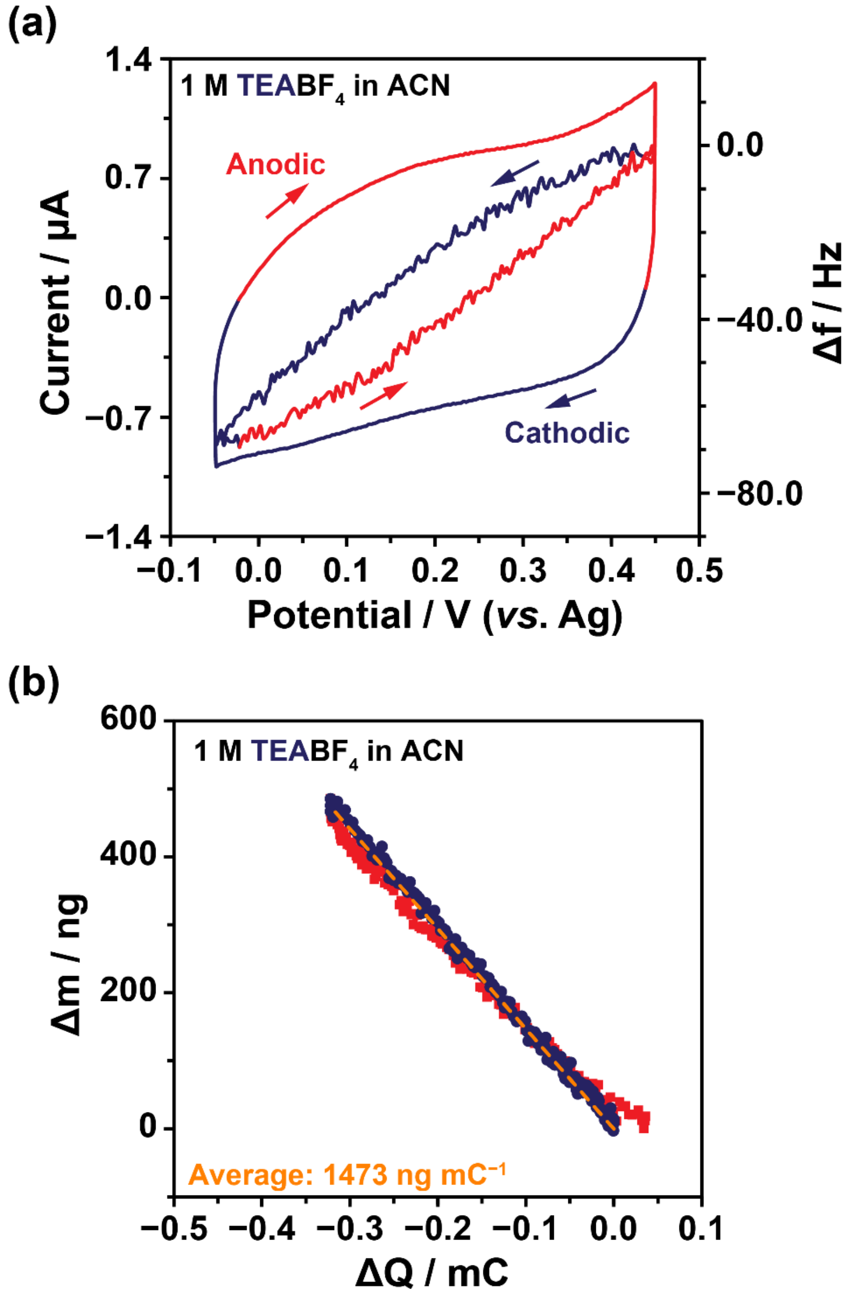


**SI Figure S17**: Repeat EQCM experiment for $Cu_3(HHTP)_2$ with 1 M $TEABF_4$ in acetonitrile electrolyte. (a) CV and EQCM frequency response obtained at a scan rate of 1 mV $s^{-1}$ in the potential range from −0.05 to +0.45 V *vs.* Ag. The EQCM cell was assembled with a $Cu_3(HHTP)_2$-coated quartz working electrode, platinum wire counter electrode, and Ag pseudo-reference electrode. (b) Plot of electrode mass change, calculated from the frequency response shown in (a), against accumulated charge. The frequency response and mass change are considered separately for cathodic (blue) and anodic (red) polarisations. The dashed line (orange) shows the average mass change during the full CV experiment. The OCP of the cell used to produce the data was +0.18 V *vs.* Ag. This data, together with the EQCM data for this system presented in Figure 3 of the Main Text, was used to obtain the error of the EQCM measurements.

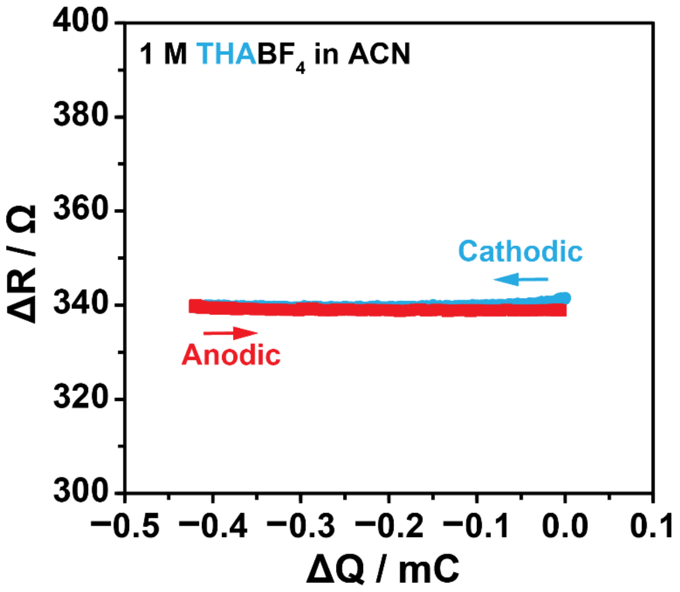


**SI Figure S18**: Motional resistance (ΔR) *vs*. charge (ΔQ) plot from electrochemical quartz crystal microbalance cell assembled with a $Cu_3(HHTP)_2$-coated quartz working electrode, platinum wire counter electrode, a Ag pseudo-reference electrode, and 1 M $THABF_4$ in acetonitrile as the electrolyte. This shows that there was negligible change in motional resistance during CV cycling, suggesting a homogenous and rigid coating of $Cu_3(HHTP)_2$ on the surface of the quartz crystal that did not detach from the crystal during electrochemical testing in this electrolyte.

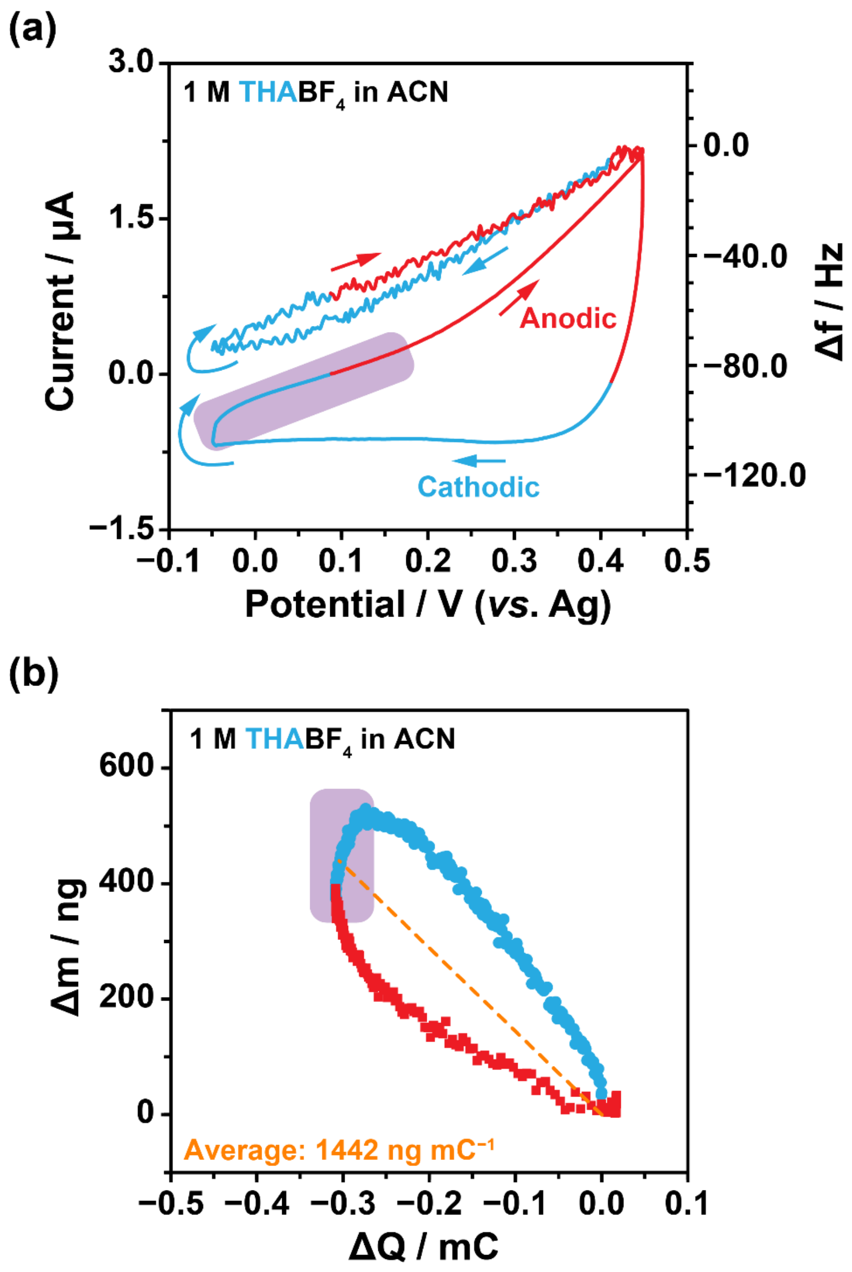


**SI Figure S19**: Repeat EQCM experiment for $Cu_3(HHTP)_2$ with 1 M $THABF_4$ in acetonitrile electrolyte. (a) CV and EQCM frequency response obtained at a scan rate of 1 mV $s^{-1}$ in the potential range from −0.05 to +0.45 V *vs.* Ag. The EQCM cell was assembled with a $Cu_3(HHTP)_2$-coated quartz working electrode, platinum wire counter electrode, and Ag pseudo-reference electrode. (b) Plot of electrode mass change, calculated from the frequency response shown in (a), against accumulated charge. The frequency response and mass change are considered separately for cathodic (blue) and anodic (red) polarisations. The dashed line (orange) shows the average mass change during the full CV experiment. The abnormal mass drop is highlighted in violet. The OCP of the cell used to produce the data was +0.21 V *vs.* Ag. This data, together with the EQCM data for this system presented in Figure 5 of the Main Text, was used to obtain the error of the EQCM measurements.

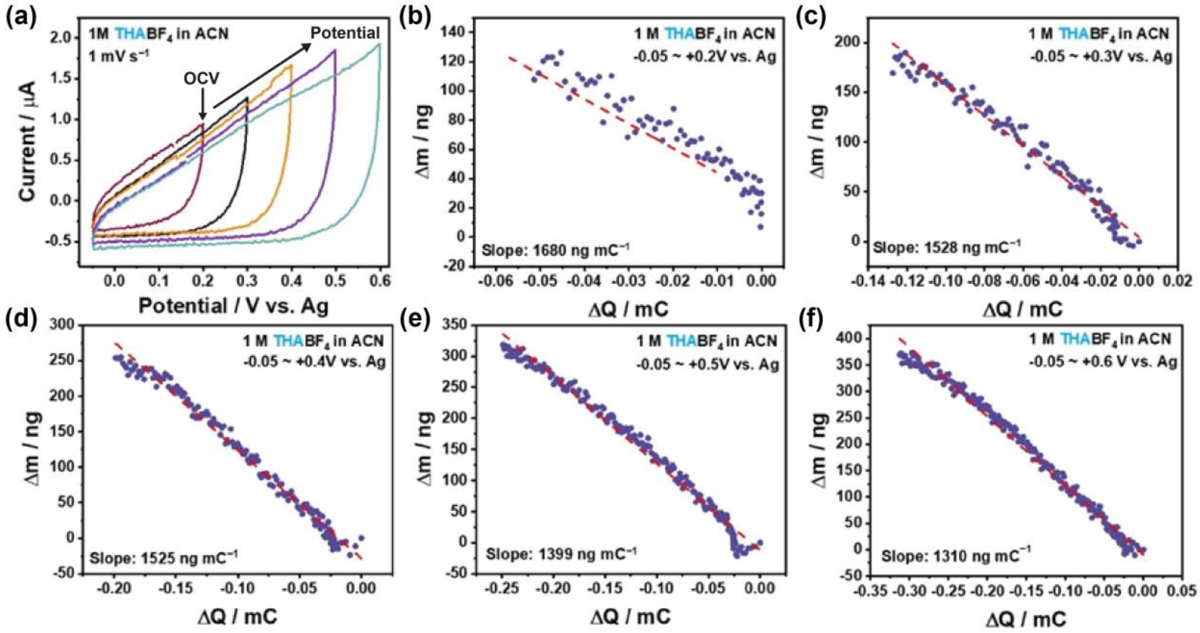


**SI Figure S20**: (a) CV data and (b) – (f) Δ*m*−Δ*Q* plots obtained from an EQCM cell assembled with a $Cu_3(HHTP)_2$ working electrode and 1 M $THABF_4$ in acetonitrile electrolyte as the potential window is gradually increased. In these experiments, the negative potential limit was fixed at −0.05 V *vs*. Ag, and the positive potential limit was gradually increased from +0.2 V *vs*. Ag (OCP) to +0.6 V *vs*. Ag. This gives a variation in the overall potential window from 0.25 V to 0.65 V. CV data were obtained at a scan rate of 1 mV $s^{-1}$, and Δ*m*−ΔQ plots were calculated from the frequency response of the quartz crystal during charging. This shows that the asymmetric charging behavior remains unchanged as the potential window is increased, and that the increase in mass with negative accumulated electronic charge is consistent between different potential windows. This suggests a constant and potential window-independent cation-dominated charge storage mechanism. It is worth noting that the molecular weight changes for lower potential windows are also comparable (150 – 160 g $mol^{-1}$), while the slope decreases at higher potential windows (to approx. 130 g $mol^{-1}$). This may be due to increased charge consumption from irreversible processes that occur at higher potentials, leading to charge consumed that does not contribute to double-layer charging and the mass change, therefore decreasing the slope of Δ*m*−Δ*Q*.

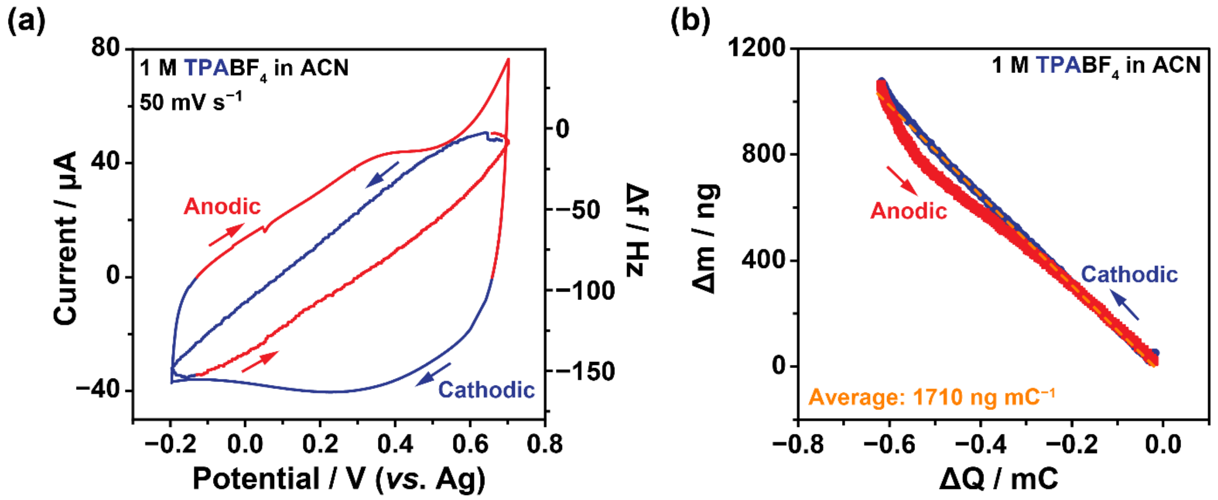


**SI Figure S21**: EQCM experiment for $Cu_3(HHTP)_2$ with 1 M $TPABF_4$ in acetonitrile electrolyte. (a) CV and EQCM frequency response obtained at a scan rate of 50 mV $s^{-1}$ in the potential range from −0.2 to +0.7 V *vs.* Ag. The EQCM cell was assembled with a $Cu_3(HHTP)_2$-coated quartz working electrode, platinum wire counter electrode, and Ag pseudo-reference electrode. A faradaic response is observed at high positive potentials due to instabilities of the EQCM cell and electrode material at these potentials. (b) Plot of electrode mass change, calculated from the frequency response shown in (a), against accumulated charge. The frequency response and mass change are considered separately for cathodic (blue) and anodic (red) polarisations. The dashed line (orange) shows the average mass change during the full CV experiment. The OCP of the cell used to produce the data was +0.20 V *vs.* Ag.

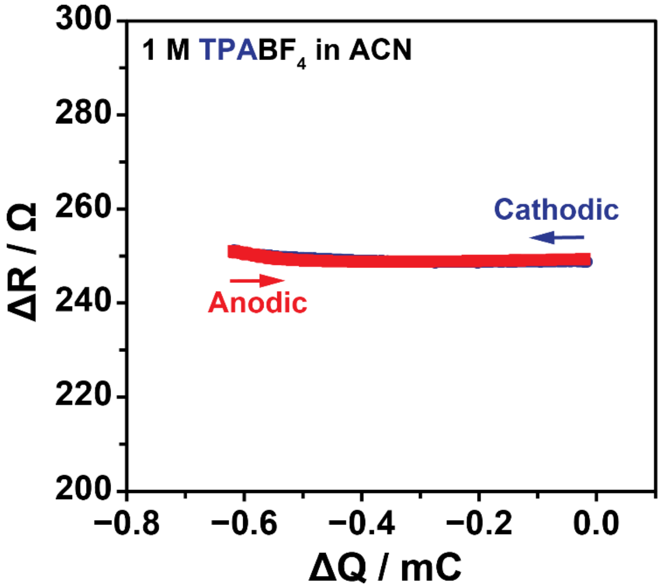


**SI Figure S22**: Motional resistance (ΔR) *vs*. charge (ΔQ) plot from the electrochemical quartz crystal microbalance cell assembled with a $Cu_3(HHTP)_2$-coated quartz working electrode, platinum wire counter electrode, a Ag pseudo-reference electrode, and 1 M $TPABF_4$ in acetonitrile as the electrolyte. This shows that there was negligible change in motional resistance during CV cycling, suggesting a homogenous and rigid coating of $Cu_3(HHTP)_2$ on the surface of the quartz crystal that did not detach from the crystal during electrochemical testing in this electrolyte.

# SI Additional Details

## Details of EQCM Calculations

According to the Sauerbrey equation, a change in mass of a quartz crystal ($\Delta m$) results in a corresponding change in the resonance frequency of the crystal ($\Delta f$)[5,6]:

$$\Delta f = \frac{-2f_o^2 \Delta m}{A(\mu_q \rho_q)^{\frac{1}{2}}}$$

Where $f_0$ is the resonant frequency of the quartz crystal (9 MHz for the quartz crystal used in this work), $A$ is the piezoelectrically active surface area of the crystal, $\mu_q$ is the shear modulus ($2.947 \times 10^6$ N $cm^{-2}$), and $\rho_q$ is the density of the quartz crystal (2.648 g $cm^{-3}$). Overall, the sensitivity of EQCM to mass change was equal to 0.1834 Hz $ng^{-1}$ $cm^{-2}$ at 20 °C in this work. The sensitivity factor of the coated quartz was also verified by performing a copper deposition experiment conducted in 0.01 M $CuSO_4$ with 0.5 M $H_2SO_4$ electrolyte. In this experiment, the sensitivity factor was calculated to be 5.43 ng $Hz^{-1}$ $cm^{-2}$, in alignment with the reported theoretical value of 5.45 ng $Hz^{-1}$ $cm^{-2}$.[7,8]

The theoretical mass change ($\Delta m$) induced by adsorption/desorption of electrolyte species during cycling can be related to the charge ($Q$) passed through the electrode using Faraday's law:

$$\Delta m = \frac{Q M_W}{nF}$$

where $M_W$ is the net molecular weight of the electrolyte species adsorbed (g $mol^{-1}$), $n$ is the valence of the ions adsorbed/desorbed (for $TEA^+$ and $BF_4^-$, $n = 1$) and $F$ is the Faraday constant (96485 C $mol^{-1}$). Therefore, the net molecular weight of the electrolyte species adsorbed or desorbed can be calculated from the slope of the $\Delta m$–$\Delta Q$ plot using the following equation:

$$\frac{M_W}{nF} = \frac{\Delta m}{Q}$$

From this, the electrolyte species that are adsorbed and desorbed upon electrochemical cycling can be deduced.

## References


(1) Gittins, J. W.; Balhatchet, C. J.; Fairclough, S. M.; Forse, A. C. Enhancing the Energy Storage Performances of Metal–Organic Frameworks by Controlling Microstructure. *Chem Sci* **2022**, *13* (32), 9210–9219. https://doi.org/10.1039/D2SC03389E.

(2) Thommes, M.; Kaneko, K.; Neimark, A. V.; Olivier, J. P.; Rodriguez-Reinoso, F.; Rouquerol, J.; Sing, K. S. W. Physisorption of Gases, with Special Reference to the Evaluation of Surface Area and Pore Size Distribution (IUPAC Technical Report). *Pure and Applied Chemistry* **2015**, *87* (9–10), 1051–1069. https://doi.org/10.1515/PAC-2014-1117.

(3) Gittins, J. W.; Balhatchet, C. J.; Chen, Y.; Liu, C.; Madden, D. G.; Britto, S.; Golomb, M. J.; Walsh, A.; Fairen-Jimenez, D.; Dutton, S. E.; Forse, A. C. Insights into the Electric Double-Layer Capacitance of Two-Dimensional Electrically Conductive Metal–Organic Frameworks. *J Mater Chem A Mater* **2021**, *9* (29), 16006–16015. https://doi.org/10.1039/D1TA04026J.

(4) Shin, S.-J.; Gittins, J. W.; Golomb, M. J.; Forse, A. C.; Walsh, A. Microscopic Origin of Electrochemical Capacitance in Metal–Organic Frameworks. *J Am Chem Soc* **2023**, *145*, 57. https://doi.org/10.1021/JACS.3C04625/ASSET/IMAGES/LARGE/JA3C04625_0007.JPEG.

(5) Sauerbrey, G. Z. The Use of Quartz Oscillators for Weighing Thin Layers and for Microweighing. *Z. Phys.* **1959**, *155*, 206–222.

(6) Chang, H.-C.; Cheng, T.-J.; Chen, R.-J. EQCM Studies of Paraquat on Gold Electrode Modified with Electropolymerized Film. *Electroanalysis* **1998**, *10* (18), 1275–1280. https://doi.org/10.1002/(SICI)1521-4109(199812)10:18<1275::AID-ELAN1275>3.0.CO;2-X.

(7) Yang, J. C.; Park, J. Molecular Imprinting of Bisphenol A on Silica Skeleton and Gold Pinhole Surfaces in 2D Colloidal Inverse Opal through Thermal Graft Copolymerization. *Polymers 2020, Vol. 12, Page 1892* **2020**, *12* (9), 1892. https://doi.org/10.3390/POLYM12091892.

(8) Jin, Y. J.; Park, J. QCM-Based HCl Gas Sensors Using Spin-Coated Aminated Polystyrene Colloids. *Polymers 2020, Vol. 12, Page 1591* **2020**, *12* (7), 1591. https://doi.org/10.3390/POLYM12071591.

(9) Shulga, Y. M.; Vasilets, V. N.; Kiryukhin, D. P.; Voylov, D. N.; Sokolov, A. P. Polymer Composites Prepared by Low-Temperature Post-Irradiation Polymerization of C2F4 in the Presence of Graphene-like Material: Synthesis and Characterization. *RSC Adv* **2015**, *5* (13), 9865–9874. https://doi.org/10.1039/C4RA09074H.
